# Structural and computational design of a SARS-CoV-2 spike antigen with improved expression and immunogenicity

**DOI:** 10.1126/sciadv.adg0330

**Published:** 2023-06-07

**Authors:** James A. Williams, Marco Biancucci, Laura Lessen, Sai Tian, Ankita Balsaraf, Lynn Chen, Chelsy Chesterman, Giulietta Maruggi, Sarah Vandepaer, Ying Huang, Corey P. Mallett, Ann-Muriel Steff, Matthew James Bottomley, Enrico Malito, Newton Wahome, Wayne D. Harshbarger

**Affiliations:** GSK, Rockville, MD, USA.

## Abstract

Severe acute respiratory syndrome coronavirus 2 (SARS-CoV-2) variants of concern challenge the efficacy of approved vaccines, emphasizing the need for updated spike antigens. Here, we use an evolutionary-based design aimed at boosting protein expression levels of S-2P and improving immunogenic outcomes in mice. Thirty-six prototype antigens were generated in silico and 15 were produced for biochemical analysis. S2D14, which contains 20 computationally designed mutations within the S2 domain and a rationally engineered D614G mutation in the SD2 domain, has an ~11-fold increase in protein yield and retains RBD antigenicity. Cryo–electron microscopy structures reveal a mixture of populations in various RBD conformational states. Vaccination of mice with adjuvanted S2D14 elicited higher cross-neutralizing antibody titers than adjuvanted S-2P against the SARS-CoV-2 Wuhan strain and four variants of concern. S2D14 may be a useful scaffold or tool for the design of future coronavirus vaccines, and the approaches used for the design of S2D14 may be broadly applicable to streamline vaccine discovery.

## Teaser

A spike antigen is designed with high expression that elicits a nAb response in mice effective against the Wuhan strain and VoCs.

## INTRODUCTION

Severe acute respiratory syndrome coronavirus 2 (SARS-CoV-2), the causative agent for coronavirus disease 2019 (COVID-19), was first identified in Wuhan, China before spreading globally and being declared a pandemic in March 2020. With more than 613 million confirmed cases globally, resulting in more than 6.5 million deaths, COVID-19 remains a notable global health burden. Vaccines, such as mRNA-1273 (Moderna), BNT162b2 (Pfizer/BioNTech), and Ad.26.COV2.S (Janssen), are all based on the sequence of the original Wuhan-Hu-1 strain of the spike fusion glycoprotein (S), engineered to remain in the prefusion state, which is the primary target of neutralizing antibodies (nAbs). However, the efficacies of these first-generation vaccines are diminished against newly circulating variants of concern (VoCs) such as Alpha (B.1.1.7), Beta (B.1.351), Delta (B.1.617.2), and Omicron (BA.1, BA.2, BA.4/5, XBB, and BQ.1.1), which evade nAbs due to mutations in the S protein (*1*–*8*). Although booster vaccines have been developed to match S protein sequences of circulating variants (*9*–*12*), there is no guarantee that these updated vaccines will protect against future strains of the virus (*7*, *8*, *13*–*15*). Therefore, there is a need for the development of vaccine antigens that can elicit Abs that are more efficient in neutralizing future variants, thus providing broader and/or longer-lasting protection.

The S glycoprotein is composed of S1 and S2 subunits that mediate host-cell attachment to initiate virus-cell fusion (*16*, *17*). The S1 subunit comprises the N-terminal domain (NTD) and the receptor binding domain (RBD) with subdomains SD1 and SD2 undergoing hinge-like motions that promote RBD movement between closed and opened conformations (*18*, *19*). Engagement of the RBD open conformation with human angiotensin-converting enzyme 2 receptor (hACE2) triggers a large-scale conformational rearrangement in the S2 domain, which contains the fusion machinery necessary to mediate viral fusion and infectivity (*17*, *20*). A large portion of the nAb response elicited during natural infection or vaccination is directed toward epitopes spanning the RBD (*21*–*24*). Epitopes in less accessible regions of the RBD are more conserved among circulating coronaviruses, consistent with Abs targeting these regions being able to cross-neutralize other coronaviruses (*21*, *25*, *26*). Several groups have developed adjuvanted RBD-only vaccines based on the monomeric or multivalent display of the RBD, with several human phase 1/2 and phase 3 trials showing a safe and immunogenic response, thus making the RBD, or S in the RBD open state, an attractive vaccine antigen (*27*–*36*).

Engineering of the S protein to remain in the prefusion state was accomplished through the rational, structure-guided insertion of two proline residues, K986P and V987P (named S-2P), which are located between the heptad repeat region 1 (HR1) and the central helix (CH) domain (*20*). S-2P was later modified to have higher thermostability and greater protein expression through the incorporation of additional prolines (F817P, A892P, A899P, and A942P) (referred to as S-6P or HexaPro), which are located between the fusion peptide proximal region, HR1, and the CH domain (*37*). Similar structure-based approaches have also been instrumental in the design of vaccine antigens for other pathogens such as the respiratory syncytial virus fusion protein F, metapneumovirus fusion protein F, influenza hemagglutinin (HA), and HIV-1 envelope (*38*–*43*). However, the identification of an antigen with the preferred qualities (i.e., conformation, stability, and expression) often requires testing hundreds of single-point mutations followed by rounds of combinatorial design which can lead to many failures.

Data-driven computational approaches have the potential to reveal mutable space within a protein’s structure that may not be obvious in a rational-based setting and assist antigen design by identifying sequences that yield desired protein characteristics (*44*–*48*). One such computational method is PROSS (Protein Repair One-Stop Shop), which is a design algorithm using evolutionary information in multisequence alignments to focus the protein design search on residues that are functional in nature (*44*). Combined with the Rosetta design suite and energy scoring function (*49*, *50*), false positive predictions can be minimized and the number of variants that must be experimentally tested is reduced (*44*, *51*, *52*). Success has been shown for multiple enzymes and the HIV gp140 glycoprotein, where using PROSS led to enhanced protein stability and greater expression of functional protein compared to wild type (*44*, *52*, *53*).

Here, we adapted the PROSS workflow to perform antigen design based on a multisequence alignment of related coronavirus glycoproteins and incorporated symmetry protocols to uniformly mutate the S protein’s trimeric structure. The evolutionary consensus design strategy resulted in a novel prefusion S antigen that had biochemical and biophysical characteristics comparable to S-2P, but with greater expression and capable of eliciting higher levels of nAb titers against Wuhan and VoCs in mice. High-resolution cryo–electron microscopy (cryo-EM) structures of our top design, S2D14, confirm the structural integrity of the spike protein and the dynamic nature of the RBDs, thus allowing exposure of potently neutralizing epitopes. This work provides a computational and experimental workflow for improving antigen characteristics, such as expression and immunogenicity, and may be valuable for informing the design of future spike-based vaccines that can elicit broad protection against emerging and future strains of the virus.

## RESULTS

### Computational design of SARS-CoV-2 spike antigens

**Fig. 1.**
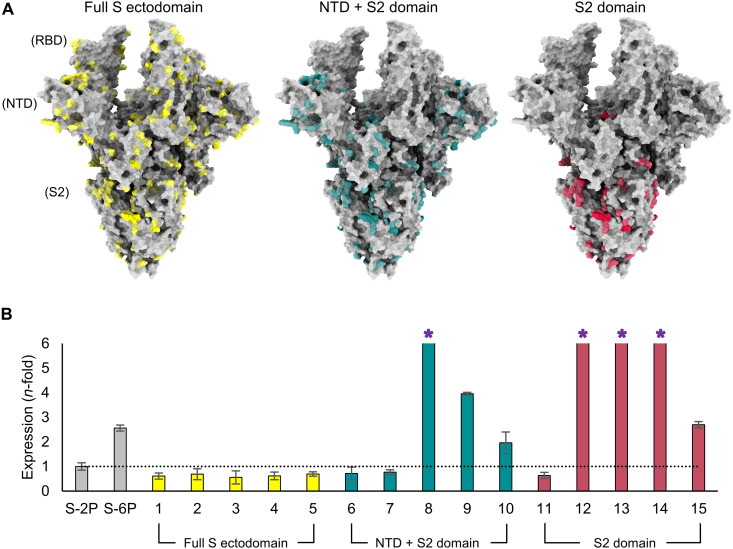
Evolutionary-based design strategy and expression levels of spike mutants. (**A**) Model of the SARS-CoV-2 spike (S) protein structure with all three RBDs in the open conformation. The first design strategy (left) allowed for mutations (yellow) across the full S ectodomain. The second design strategy (center) limited the design landscape to the NTD + S2 domain (mutations in teal). The third design strategy (right) only modified the S2 domain and mutations are colored red. (**B**) Protein expression level (determined by biolayer interferometry using anti-HIS biosensors) of spike mutants normalized to S-2P (shown by dotted line) and grouped according to domain-specific design strategies in (A). S-6P (HexaPro) was used as an additional comparator. Error bars were determined from duplicate measurements, and asterisks indicate designs where expression exceeded the assay’s limit of quantitation.

The evolutionary consensus design workflow combines three distinct steps, as previously described (*44*). First, we generated a multisequence alignment of 500 nonredundant spike protein sequences from various betacoronavirus lineages (lineages *A* to *D*), which we obtained from the BLAST database (data S1) (*54*), allowing for the identification of residues with natural variation. Next, Rosetta atomistic design simulations (*50*) were used to curate which set(s) of single-point mutations could be applied to the above-identified residues to obtain unique spike designs with predicted lower free energy (i.e., improved stability) compared to the sequence of the target S antigen model (*20*). To generate an initial S protein model, we chose to incorporate a symmetry-based protocol (*55*) with a molecular structure that has all three RBDs in the open conformation which places the RBDs away from the neighboring S protomers (Fig. 1) (see Materials and Methods). We believed that this would prevent the possibility of designs being stabilized in the three-RBD closed conformation, therefore ensuring that important neutralizing epitopes remained accessible. Specifically, our starting model contained three mutations: (i and ii) the S-2P di-proline mutations (K986P, V987P) which were used to ensure that the prefusion conformation of the spike was maintained (*20*) and (iii) a D614G mutation, located within the SD2 domain, which was included because it was an overrepresented mutation in the above sequence alignments from circulating strains at the time of this study (*56*, *57*). Modification of these mutations was prohibited during design simulations and is therefore conserved across all designed sequences (data S2). In the final design step, Rosetta combinatorial sequence optimization was used to generate constructs with energy profiles more favorable (lower energy) than the initial S antigen model (fig. S1).

Because of the difficulty of modeling large and dynamic proteins such as S, and facing the substantial sequence diversity in coronavirus families, three independent strategies were used for this final design step to increase the probability of identifying sequences that yield desirable protein characteristics. The first strategy incorporated mutations across the full S ectodomain, the second strategy limited the design space to the NTD + S2 domain, and the third strategy further restricted the mutations to the S2 domain (Fig. 1A). A total of 36 designs (12 constructs per strategy) were created, where each design contained a total of 28 to 141 mutations (full S ectodomain), 17 to 103 mutations (NTD + S2), or 12 to 25 mutations (S2 domain). To narrow the candidate pool for in vitro analysis, the 12 designs per strategy were placed into five groups based on phylogenetic analysis, and one representative S antigen from each of these groups was selected for production and purification, followed by biochemical and biophysical characterization (fig. S1, A to C).

### Expression, antigenicity, and stability of spike designs

Genes containing sequences for each of the 15 selected constructs were cloned into a mammalian expression vector and tested for expression in human embryonic kidney (HEK) 293 cells. Among the 15 designs selected, 7 (three for the NTD + S2 and four for the S2 domain only) displayed expression levels in cell culture supernatants that exceeded S-2P, with five of those designs (numbers 8, 9, 12, 13, and 14) also having expression levels in supernatant higher than S-6P (Fig. 1B). Specifically, design 8 (NTD + S2 domain) and designs 12, 13, and 14 (S2 domain only) displayed the largest increase in nonpurified protein expression relative to S-2P (>6-fold) or S-6P (>3-fold) and exceeded the upper limit of quantification of the assay, while design 9 (NTD + S2 domain) had an ~4-fold and ~1.5-fold increase compared to S-2P and S-6P, respectively. The expression levels of designs targeting the full S ectodomain were considerably lower than those detected for S-2P (~1.5-fold) or S-6P (~4-fold). This is consistent with observations that, despite the high tolerance for variability within the RBD, many mutations can result in deleterious effects on protein expression (*58*). Designs 6 and 7 (NTD + S2 domain) and design 11 (S2 domain) also had expression levels lower than S-2P. On the basis of these results, full S ectodomain designs, as well as designs 6, 7, and 11, were not considered for further biochemical or biophysical characterization, leaving seven designs for further analysis.

Considering that the majority of the most potent SARS-CoV-2 nAbs isolated to date target epitopes spanning the surface of the RBD, surface plasmon resonance (SPR) was used to determine whether incorporated mutations within the NTD or S2 domain inadvertently altered the presentation or accessibility of key RBD epitopes. CR3022, which requires at least two RBDs in the open state, S309, which recognizes open or closed RBDs, and the hACE2 receptor, which binds a single RBD in the open conformation, were all found to bind designs 8, 9, and 10 (NTD + S2 domain) as well as designs 12, 13, and 14 (S2 domain) with equilibrium dissociation binding constants (*K*_d_ values) in the picomolar range (Table 1, fig. S2, and table S1). These values were comparable to those found for binding to S-2P, thus indicating no structural disruptions to these RBD epitopes.

**Table 1.** Thermal stability and binding affinity measurements for spike protein designs. The T_m_ values are the average of triplicate measurements with the exception of design 10, which was limited by protein quantity. For binding affinity values, the average and SD of triplicate measurements are shown with the exception of design 15, which was not measured because of limited protein quantity. The binding of design 10 to VRC-118 was limited to a single measurement because of limited protein quantity. NM, not measured; NB, no binding.

Designed mutant	Design strategy	T_m_1 (°C)	T_m_2 (°C)	Binding affinity, *K*_d_ (pM)				
				ACE2	CR3022	S309	VRC-118	VRC-112
8	NTD + S2	46.38	76.34	160 ± 14	27 ± 4	19 ± 16	9.7 ± 0.4	NB
9	NTD + S2	48.35	79.65	150 ± 5	370 ± 240	44 ± 6	2.9 ± 0.5	NB
10	NTD + S2	46.99	77.44	44 ± 4	2.4 ± 3.3	0.16 ± 0.07	1.9	NB
12	S2 domain	43.72	86.86	100 ± 2	9.0 ± 0.9	11 ± 2	14 ± 8	NB
13	S2 domain	43.75	77.96	65 ± 1	2.2 ± 1.1	20 ± 3	11 ± 1	NB
14	S2 domain	44.18	78.65	190 ± 5	110 ± 51	70 ± 14	25 ± 1	NB
15	S2 domain	44.00	86.54	NM	NM	NM	NM	NM
S-2P		44.13	77.58	200 ± 18	58 ± 26	70 ± 0	42 ± 5	53 ± 4

Next, the binding of monoclonal antibodies (mAbs) VRC-118 and VRC-112, which target the NTD and S2 domains, respectively, was tested as these domains have also been shown to contribute to neutralization breadth and potency (*21*, *22*, *59*–*61*). All the designs tested were found to bind VRC-118 with picomolar affinity and with a similar *K*_d_ as S-2P. However, binding to VRC-112 was abolished for all designs, revealing that the S2 domain mutations do disrupt at least this S2 domain epitope (Table 1, figs. S2 and S3, and table S1). Despite this result, we reasoned that further characterization was warranted on the basis of the high levels of protein expression and the confirmation of intact RBD and NTD epitopes.

To assess the impact of thermal stress, purified proteins were subjected to a differential scanning fluorimetry (DSF) assay. S-2P has previously been shown to have two distinct melting transitions (T_m_1 and T_m_2) with an increase in T_m_1 being indicative of improved stability (*37*). All three NTD + S2 designs (8, 9, and 10) had T_m_1 values greater than S-2P (T_m_1 = 44°C), with design 9 having the largest, yet modest, increase of 4.2°C (T_m_1 = 48°C) (Table 1). T_m_1 values for all four S2 domain designs (12, 13, 14, and 15) were comparable to S-2P. The T_m_2 values for most designs were also comparable to S-2P, with only designs 12 and 15 showing an ~10°C increase in melting temperature, though the impact of this improvement is not clear.

### Design 14 forms stable prefusion trimers

**Fig. 2.**
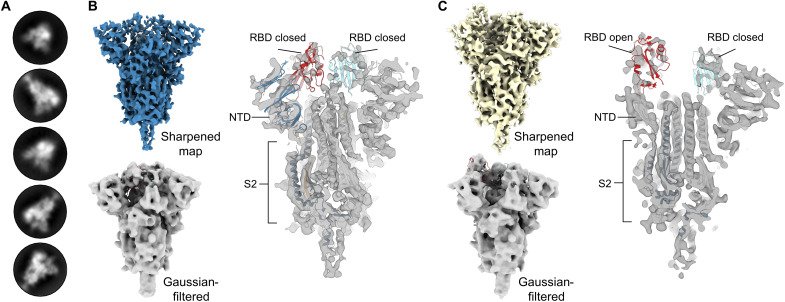
S2D14 (design 14) adopts the trimeric prefusion S conformation and exhibits RBD conformational heterogeneity. (**A**) Representative two-dimensional classes of S2 domain design S2D14 from cryo-EM micrographs, confirming the expected S protein trimers. (**B**) The cryo-EM map of S2D14 in a three-RBD closed state at 7.1-Å resolution is shown above and colored blue. A Gaussian-filtered volume with a rigid body fit of the S protein in the closed conformation [Protein Data Bank (PDB) accession number: 6VXX] is shown below the cryo-EM map and more clearly displays the three-RBD closed state. Shown on the right is a cross section of the sharpened map with a rigid body fit of the S protein in the closed conformation (PDB accession number: 6VXX). (**C**) The cryo-EM map of S2D14 with one RBD in the open conformation at 8.5-Å resolution and obtained from the same dataset as (B) is shown above and colored yellow. A Gaussian-filtered volume with a rigid body fit of the S protein in the one-RBD open state is shown below the cryo-EM map and, more clearly, displays the one-RBD open state. Shown on the right is a cross section of the EM map with a rigid body fit of the spike protein in the one-RBD open state (PDB accession number: 6VSB).

Considering the loss of binding to VRC-112 for each of the designs, we next sought to determine whether modifications had inadvertently altered the S2 domain architecture or overall protein morphology. To accomplish this task, a Glacios 200-kV electron microscope was used to quickly assess one construct from the NTD + S2 designs and one construct from the S2 domain designs. Design 9 was chosen from the NTD + S2 designs based on the high expression and thermostability; however, particles resembling the prefusion S trimer were infrequently observed on the cryo-EM micrographs (fig. S4A). This may be due to dissociation into monomers or other misfolded states during storage or vitrification, consistent with known issues of cold handling with spike proteins (*62*). From the S2 domain designs, design 14 was selected on the basis of the high level of protein expression and similar thermostability and antigenicity as S-2P. Cryo-EM micrographs of design 14 revealed the expected S protein trimers with the anticipated particle size and secondary structural features (Fig. 2A and fig. S4B).

Two distinct conformational states for design 14 were identified during three-dimensional (3D) classification: one conformation with all three RBDs in the closed state and the second conformation with a single RBD domain in the open state (Fig. 2, B and C, and fig. S5A). The refinement of each population resulted in 7.1-Å resolution (three RBDs closed) and 8.5-Å resolution (one RBD open) maps, respectively (Fig. 2, B and C, Table 2, and fig. S5B). The sampling of RBD open states is consistent with the recognition of the hACE2 receptor and mAb CR3022. Docking of previously published structures for either the closed spike trimer [Protein Data Bank (PDB) accession number: 6VXX] or a single RBD open trimer (PDB accession number: 6VSB) into the corresponding electron density maps revealed a high degree of similarity across the S2 domains for either model, indicating that engineered S2 mutations were well tolerated and did not alter the S protein morphology. At this stage, design 14 was considered our top candidate for further evaluation and renamed S2D14 for simplicity.

**Table 2.** Cryo-EM data collection and image processing statistics for S2D14. EMDB, Electron Microscopy Data Bank; PDB, Protein Data Bank; RMSD, root mean square deviation.

Data collection					
Microscope	Glacios		TitanKrios		
Voltage (kV)	200		300		
Detector	Falcon 3		Falcon 4		
Magnification	120,000		120,000		
Pixel size (Å/pixel)	0.91		0.67		
Exposure (e/Å^2^)	48		43		
No. of frames	50		42		
Defocus range (μm)	0.75–2.5		0.8–2.2		
Software	EPU		EPU		
Total micrographs	1425		6169		
**Data processing**					
Particles extracted	92,933		724,002		
After 2D classification	44,459		566,825		
	3-RBD down	1-RBD open	3-RDB down	2-RBD exposed	2-RBD open
Particles in refinement	7759	9154	33,173	47,773	87,614
Symmetry	C3	C1	C3	C1	C1
Map sharpening B-factor	−389.99	−592.139	−17.18	−5.35	−7.2
Unmasked resolution at 0.5/0.143 FSC (Å)	10/7.5	13/9.1	4.2/3.5	7.9/4.1	7.3/3.9
Masked resolution at 0.5/0.143 FSC (Å)	8.1/7.1	9.0/8.5	3.3/2.8	3.9/3.3	3.7/3.1
PDB ID			8EPN	8EPQ	8EPP
EMDB ID			28531	28533	28532
**Model statistics**					
MolProbity score			1.50	1.60	1.67
Clash score			6.70	7.20	7.58
Composition					
Amino acids			2991	2984	2939
Glycans			126	66	66
RMSD bonds (Å)			0.003	0.003	0.002
RMSD angles (°)			0.579	0.609	0.491
C-beta outliers (%)			0.00	0.00	0.00
CC (mask)			0.87	0.81	0.84
Rotamer outliers (%)			0.84	0.12	0.35
Ramachandran plot					
Favored (%)			97.34	96.75	96.21
Allowed (%)			2.66	3.21	3.65
Disallowed (%)			0.00	0.03	0.14

**Fig. 3.**
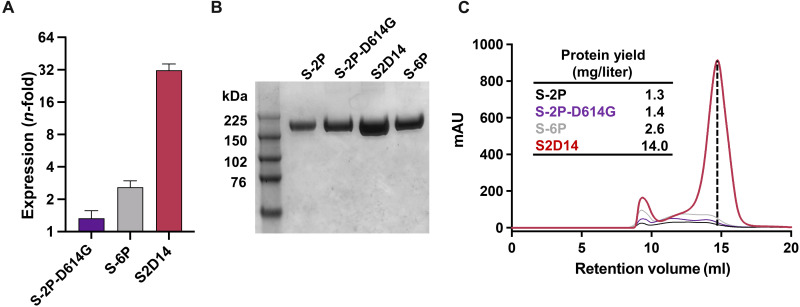
S2 domain mutations in S2D14 enhance expression of functional prefusion trimer. (**A**) Total protein expression in cell culture supernatant for S proteins showing an *n*-fold increase relative to S-2P. A total of six replicate measurements were taken, and error bars represent SD from the means. (**B**) SDS–polyacrylamide gel electrophoresis analysis of size exclusion chromatography (SEC)–purified S proteins. Relevant bands for the molecular weight ladder (lane 1) are labeled in kilodaltons. (**C**) Overlay of the SEC chromatograms for S protein antigens. The dashed vertical line indicates the peak retention volume for the S2D14 trimer. The final yield of purified S protein trimers is shown in the inset.

Next, we wanted to evaluate whether the high expression of S2D14 in HEK293 cell culture supernatants translated into higher levels of purified, functional protein compared to S-2P and S-6P. In addition, to compare the possible contribution of the D614G mutation for enhancing the expression of S-2P, an S-2P variant that contains the D614G mutation, named S-2P–D614G, was created and tested as well. Purified S2D14 protein yielded an ~11-fold increase relative to S-2P and an ~5.4-fold increase relative to S-6P (Fig. 3, A to C). The purified yield of S-2P–D614G was nearly identical to S-2P, confirming that the increased expression obtained for S2D14 is attributed to the mutations incorporated through computational design.

### Immunization with S2D14 elicits high neutralizing antibody titers

**Fig. 4.**
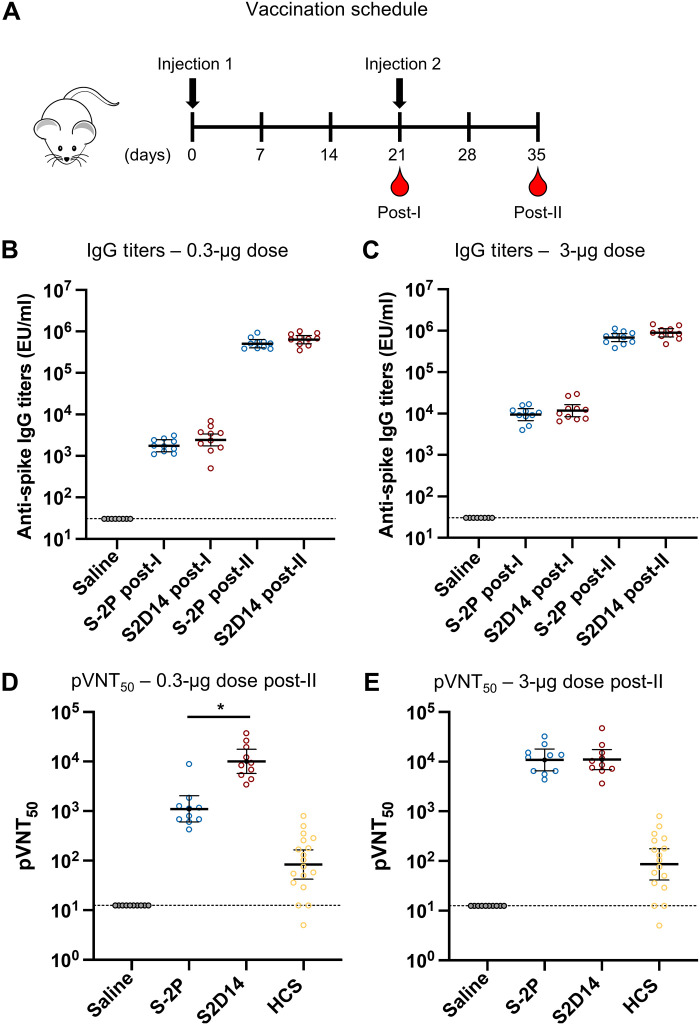
IgG and neutralizing antibody titers from mice immunized with adjuvanted S2D14 or S-2P. (**A**) Mouse immunization schedule. Mice were immunized with either a 0.3- or 3.0-μg doses containing AS03 adjuvanted S2D14 or S-2P. Serum was collected 3 weeks after the first immunization (post-I) and 2 weeks after the second immunization (post-II). (**B** and **C**) Enzyme-linked immunosorbent assay immunoglobulin G (IgG) titers of S2D14 compared to S-2P at both 0.3-μg (B) and 3.0-μg (C) doses for both post-I and post-II serum collections. Individual data with geometric mean titers (GMT) and 95% confidence intervals (CIs) are presented. (**D** and **E**) 50% pseudovirus neutralization titers (pVNT_50_) against the Wuhan strain were analyzed 2 weeks post-II at two antigen dosages, 0.3 (D) and 3.0 μg (E). Neutralizing antibody titers at either dose post-II were noticeably greater than for human convalescent sera (HCS). Individual data with GMT and 95% CIs are presented. For HCS samples, GMT and 95% CIs were calculated separately from vaccination groups in GraphPad Prism. Asterisks, *, indicate a statistically significant increase in neutralization response based on geometric mean ratio (GMR) comparisons with a two-sided 90% CI. Ratios for which the CI does not include 1 are considered statistically significant. See Materials and Methods for a description of the sera panel.

To assess the immunogenicity of S2D14 in comparison to S-2P, we immunized BALB/c mice with AS03 (oil-in-water emulsion) adjuvanted proteins at either 0.3- or 3.0-μg doses. Intramuscular injections were given on days 0 and 21, with serum collected 3 weeks post-I (first immunization) injection and two weeks post-II (second immunization) injection (Fig. 4A). Total anti-spike immunoglobulin G (IgG) titers [assessed by anti-spike enzyme-linked immunosorbent assay (ELISA)] indicated that S2D14 was immunogenic at both the 0.3- and 3.0-μg doses and that titers were boosted after the second dose (Fig. 4, B and C). Anti-spike IgG titers were comparable between S-2P and S2D14 at both post-I and post-II time points at the 0.3-μg dose and for the 3.0-μg dose (Fig. 4, B and C).

S2D14 elicited a robust neutralizing antibody response to the Wuhan strain at post-II for each vaccine dose (Fig. 4, D and E). At post-II for the 0.3-μg dose, S2D14 induced statistically significant higher neutralizing antibody titers compared to S-2P (9.1-fold increase). In addition, both S-2P and S2D14 had low neutralizing titers post-I at either dose (fig. S6), but noticeably higher neutralizing titers at either dose at post-II than for human convalescent serum (HCS) (Fig. 4, D and E) (*63*). The post-II neutralizing titers were highly similar between 0.3- and 3.0-μg doses. While it is possible that nAb titers reached saturation, the saturating dose in each assay is not known.

**Fig. 5.**
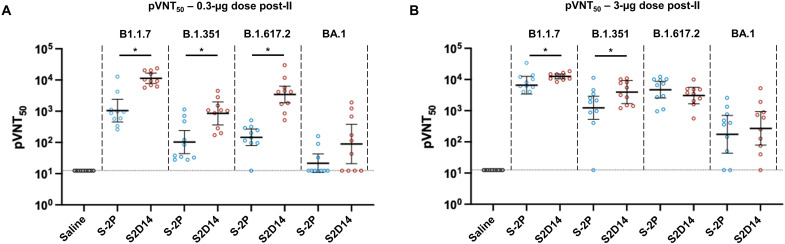
S2D14 elicits neutralizing antibody titers in mice capable of neutralizing variants of concern. (**A**) Neutralization of VoCs compared between S2D14 and S-2P at low doses for post-II serum collection. S2D14 induced statistically significant higher neutralization responses against the Alpha (B1.1.7), Beta (B.1.351), and Delta (B.1.617.2) variants compared to S-2P. (**B**) Neutralization of VoCs compared between S2D14 and S-2P at high doses for post-II serum collection. S2D14 induced statistically significant higher neutralization responses against the Alpha (B1.1.7) and Beta (B.1.351) variants compared to S-2P. Individual data with GMT and 95% CIs are presented. Asterisks, *, indicate a statistically significant increase in neutralization response based on GMR comparisons with a two-sided 90% CIs. Ratios for which the CI does not include 1 are considered statistically significant.

Next, we determined neutralizing antibody titers against VoCs that were available at the time of this study: Alpha (B1.1.7), Beta (B.1.351), Delta (B.1.617.2), and Omicron (BA.1) strains (Fig. 5, A and B). S2D14 elicited a neutralizing antibody response to all the variant strains when immunized at both the 0.3- and 3.0-μg doses but with an overall decrease in neutralizing antibody titers compared to the Wuhan strain for the Beta (~10-fold), Delta (~5-fold), and Omicron (~100-fold) variants. Compared to S-2P at post-II for the 0.3-μg dose, S2D14 induced statistically significant higher neutralizing antibody responses against the Alpha (10.8-fold increase), Beta (8.2-fold increase) and Delta (23.4-fold increase) variants (Fig. 5A). Regarding the Omicron strain, although many mice did not show measurable titers (4 out of 10 in the S2D14 group and 7 out of 10 in the S-2P group), and the difference was not statistically significant, at 0.3-μg dose, S2D14 did elicit an ~10-fold higher neutralizing antibody response than S-2P. At post-II for the 3.0-μg dose, S2D14 elicited statistically significant higher neutralizing antibody responses against the Alpha and Beta variants but were comparable to S-2P for the Delta and Omicron variants (Fig. 5B). Together, these data show that S2D14 is immunogenic and capable of eliciting high levels of IgG binding antibodies (Wuhan) and nAbs (Wuhan and VoC strains). In addition, although the total IgG titers are similar between S2D14 and S-2P after vaccination, the antibody response elicited by S2D14 at the 0.3-μg dose is superior in neutralization potency and breadth.

### Cryo-EM structures of S2D14 reveal a range of RBD conformational states

**Fig. 6.**
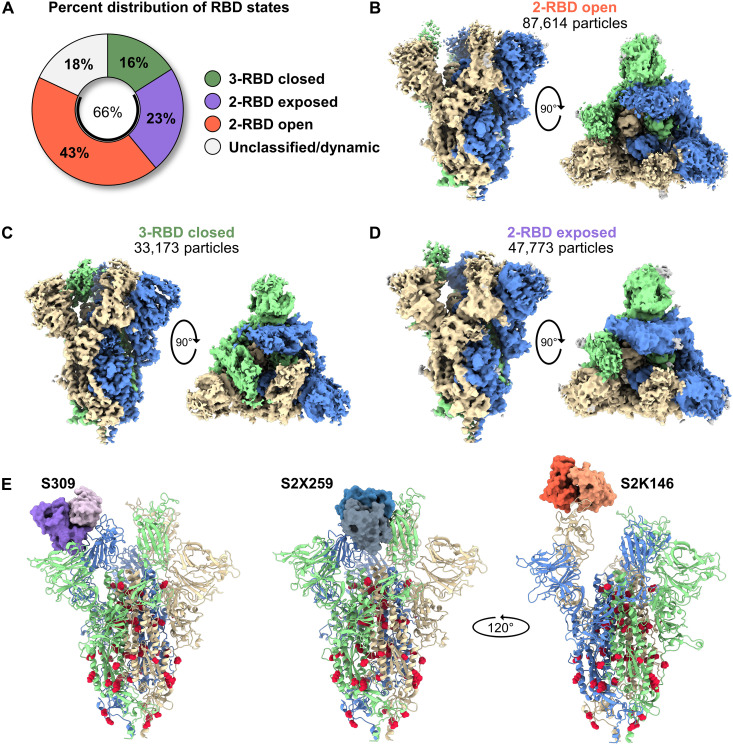
S2D14 displays RBD open conformational states and is accessible for binding RBD nAbs. (**A**) Pie chart illustrating the percent distribution of RBD conformational states. (**B**) The cryo-EM structure of S2D14 in the two-RBD open conformations (3.1-Å resolution) was the dominant population and represents 43% of total particles. (**C**) The cryo-EM structure of S2D14 in the three-RBD closed conformation (2.8-Å resolution) was the minority population with 16% of total particles. (**D**) The cryo-EM structure (3.3-Å resolution) of S2D14 in the two-RBD exposed state was representative of 23% of the total population. Eighteen percent of the particles could not be classified into a distinct conformation. Side and top views are shown for each structure, and monomers are individually colored for clarity. (**E**) Docking of Fabs of S309 (left), S2X259 (center), and S2K146 (right) onto S2D14 in the two-RBD open conformation confirms the accessibility of these broadly neutralizing RBD epitopes. S2D14 protomers are colored blue, green, or tan, and mutations are shown as red spheres.

To better understand the molecular basis for the improved breadth and immunogenicity of S2D14, a more extensive cryo-EM dataset was collected for high-resolution structural analysis using a Titan Krios 300-kV electron microscope. From a single dataset, we were able to determine the structure of S2D14 in three distinct conformations: two RBDs open (43% of particles, 3.1-Å resolution), three RBDs closed (16% of total particles, 2.8-Å resolution), and two RBDs exposed with one RBD down (23% of total particles, 3.3-Å resolution) (Fig. 6, A to D, Table 2, and fig. S7, A and B). Although this final class most closely aligned to the one-RBD open mask used during focused classification, two of the RBDs lacked clear density suggesting a state with two dynamic RBDs, each sampling an RBD open state, yet distinguishable from the two-RBD open structure (Fig. 6, B to D). Together, these conformations reveal that ~66% of particles contain at least two RBDs in the open or exposed states and are consistent with binding to hACE2 and CR3022. Docking of Fabs S309 (*64*), S2X259, or S2K146 (*25*, *26*) onto S2D14 confirms that neutralization-sensitive RBD epitopes, in particular those exposed only in an RBD open state (recognized by S2X259 and S2K146), are accessible with no apparent clashes (Fig. 6E).

To better assess the relative proportion of RBDs in the open conformation, we performed BLI experiments measuring the peak binding levels for hACE2 and CR3022 to S-2P, S-6P, S2D14, and the S-2P–D614G variant. In this assay, an increase in peak binding signal indicates greater accessibility of the RBDs (i.e., more open states) (*65*). Although the peak binding signal for S2D14 to hACE2 was greater than both S-2P–D614G and S-6P by ~2.2-fold and ~1.6-fold, respectively, S2D14 only reached 80% of the peak binding signal for S-2P (fig. S8A). The S-2P–D614G mutant showed the lowest peak binding levels, which was unexpected given previous data showing that the D614G mutation can enhance RBD exposure (*66*). A similar trend was also observed for binding to CR3022 (fig. S8B). Nonetheless, the PROSS mutations within S2D14 do appear to recover most of the loss in peak binding levels that may have occurred by the inclusion of the D614G mutation, which is consistent with the cryo-EM analysis showing a mix of open and closed RBD states.

**Fig. 7.**
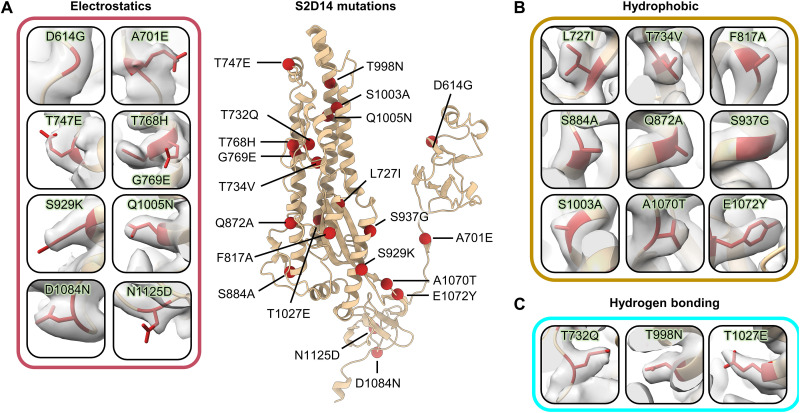
PROSS mutations incorporated in S2D14 are well resolved by cryo-EM. A single protomer of S2D14 residues 600 to 1147 is shown in the center in ribbon representation with mutations indicated as red spheres. Mutations are grouped on the basis of changes in either (**A**) electrostatic complementarity, (**B**) alterations in hydrophobicity, or (**C**) the introduction of hydrogen bonds. Residue side chains are depicted as sticks and cryo-EM density is shown as a transparent surface.

**Fig. 8.**
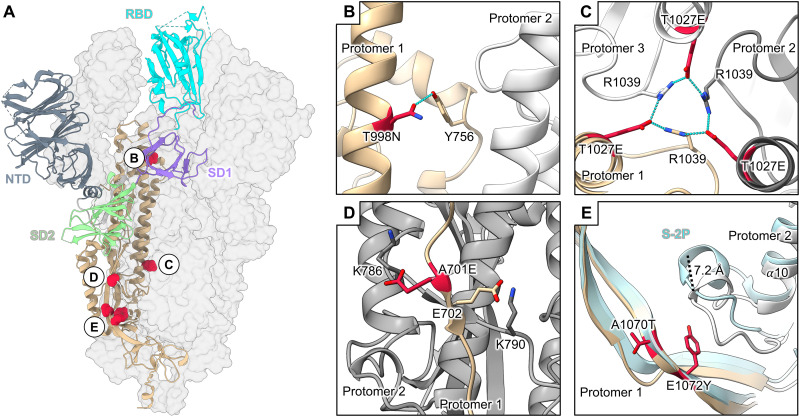
Mutations within S2D14 that may influence S2 domain stability. (**A**) S2D14 is shown with two protomers as transparent surfaces, and a single protomer is depicted as a cartoon with domains colored as labeled in the figure. Mutations that may stabilize the S2 domain are colored red and shown as spheres. (**B**) A T998N mutation in the CH domain introduces a hydrogen bond with Y756 within the same protomer. (**C**) A T1027E mutation in the S2 core forms an interprotomer hydrogen bond network between R1039 residues. (**D**) An A701E mutation creates a negative patch at the S1/S2 linker region in proximity to a positively charged lysine residue K786 on an adjacent protomer. (**E**) The E1072Y mutation creates a hydrophobic surface that displaces an adjacent loop away from the trimer interior by ~7.2 Å. The positioning of this loop for S-2P is shown for comparison (light blue).

The 23 total mutations (20 PROSS designed mutations, D614G, K986P, and V987P) present in S2D14 are all well resolved with clear side chain densities (Fig. 7, A to C). Analysis of the structures provides molecular details for the three main types of mutations incorporated by the evolutionary consensus design: (i) improvement of complementary electrostatic potential between interprotomer contact regions (Fig. 7A), (ii) optimization of hydrophobicity and van der Waals (VDW) contacts (Fig. 7B), and/or (iii) introduction of hydrogen bond interactions (Fig. 7C). Although the minimal number of mutations that are necessary to have the same properties as S2D14 was not explored, the analysis of the structure does allow for speculation of how these mutations may influence local interactions and trimer stability. For example, the mutation T998N introduces a hydrogen bond between the asparagine oxygen with the side chain of Y756, located on the same protomer, while the mutation T1027E within the CH forms a hydrogen bonding network between the E1027 side chain and R1039 of an adjacent S protomer (Fig. 8, A to C). Notably, several hydrogen bonds are also lost because of mutations such as T734V, S1003A, and Q1005N (fig. S9, A and B). However, as shown by DSF, the overall thermostability of S2D14 is the same as S-2P, suggesting that this loss of energy is well compensated not only by the formation of additional hydrogen bonds but also by the VDWs and electrostatic mutations that are introduced. One example of the addition of an electrostatic interaction is the A701E mutation, which positions the E701 side chain within ~5 Å of the K786 side chain of an adjacent S protomer and creates a complementary charged surface (Fig. 8D and fig. S9, C to E).

Superposition of the S2 domain (residues 821 to 1147) of S2D14 in the two-RBD open conformation with S-2P reveals a root mean square deviation of 1.6 Å over all C_α_ atoms (fig. S10). Despite this high structural similarity, there is a marked difference in the positioning of the loop connecting the α10 and α11 helices (residues G885 to Q895) near mutations A1070T and E1072Y located on the adjacent S protomer. These mutations reduce the overall charge of the interface between these two S protomers, creating a more hydrophobic patch that displaces the loop connecting α10 and α11 by 7.2 Å (measured from the C_α_ atom of residue A890) (Fig. 8E and fig. S11, A to E). Of note, the modification of this loop with an A892P mutation has been shown to provide an increase in protein expression and to stabilize the trimeric S protein (*37*, *67*). Although other S2 domain mutations, such as those that are surface-exposed, may also influence trimer expression and/or solubility, they are not easily rationalized by the structure and further experiments performing single-point mutations or reversions would be necessary to fully understand individual contributions.

## DISCUSSION

The emergence of SARS-CoV-2 variants has heightened concerns about the efficacy of currently approved vaccines, which are based on the prefusion stabilized S protein antigen, S-2P (*1*–*4*, *68*). Taking a computational approach starting with S-2P plus the inclusion of a predominant D614G mutation and modeled to have three RBDs in the open conformation, we used evolutionary consensus design for the in silico optimization of trimeric S antigens. These antigens were then biochemically, biophysically, structurally, and immunogenically characterized leading to the selection of design 14, named S2D14, which yielded an ~11-fold improvement in purified protein expression from HEK293 cells compared to S-2P. Immunization of mice with S2D14 resulted in higher levels of neutralizing antibody titers against Wuhan and VoC strains. Although we could not assess this experimentally, the increased expression yield suggests a greater stability of the protein in solution and offers a potential mechanism for the improved quality of the antibody response through better preservation of properly folded protein in mice after injection. Future studies should aim to experimentally compare the long-term stability between S2D14 and S-2P.

Cryo-EM structures revealed that roughly two-thirds of the S2D14 particles have either one or more RBDs in an open or exposed state, and an assessment of binding to hACE2 and CR3022 confirmed that the dynamic RBDs are not hindered by the incorporated mutations. The mutations found in S2D14 reflect strategies that would typically be used in rational-based approaches, such as improving VDW contacts, adding hydrogen bond interactions, and the insertion of complementary electrostatics (*69*). Although many modifications to the S protein have been reported in the literature to improve expression and thermostability (*70*), none of the mutations incorporated in S2D14 have been reported, providing previously unrecognized chemical space, and improving our understanding of how spike may be modified. Therefore, the advantages of the evolutionary-based strategy used here are that mutable space can be explored, which may not be obvious from a rational approach, and unique combinations of mutations that may improve protein folding and expression can be rapidly determined. In addition, since computational energetics guide the assessment, a more restricted set of constructs will likely be generated for testing in vitro, as opposed to often much larger numbers of designs if a rational approach was taken. One drawback, however, is that the computationally determined sequences contain a large number of mutations per design (20 for S2D14 not including D614G, K986P, and V987P) and the individual contribution for each mutation is not clear. Some mutations may be providing the opposite effect than what is desired. Another consideration is that other widely adopted strategies used to arrest the prefusion conformation of glycoproteins, such as incorporating non-natural disulfide bonds and helix-capping prolines, are unlikely to be inserted by an evolutionary-based approach since the design landscape is influenced by sequences circulating in nature which must contain fusion-competent S glycoproteins. Therefore, the engineering of an antigen with the desired characteristics (i.e., expression, stability, and immunogenicity) may benefit from the incorporation of both evolutionary consensus design and rational-based strategies.

Despite the development of bivalent vaccines, variants continue to emerge that challenge vaccine efficacy and durability (*71*, *72*), highlighting an urgent need for improved vaccines capable of broad protection again VoCs. Some strategies taken to improve protective efficacy have included focusing the immune response toward the RBD through multivalent display of the RBD on nanoparticles, or through the modification of the RBD sequence (*27*, *28*). In the case of nanoparticles, mosaic display of RBDs from diverse sarbecoviruses was capable of neutralizing SARS-CoV-2 variants, including Omicron, and was protective against challenges with SARS-2 Delta variant and SARS-1 in non-human primates (*27*). Alternatively, modification of the RBD sequence was shown to focus the immune response to potently neutralizing epitopes and to elicit neutralizing titers greater than the unmodified RBD in mice (*28*).

Aside from the RBD, vulnerable epitopes have also been described for the NTD, SD1, and S2 domains (*21*, *22*, *59*, *60*, *73*–*79*). The S2 domain, in particular, is highly conserved among coronaviruses, and it was found that non–SARS-CoV-2–exposed individuals had IgG that bound the CoV-2 S2 domain but not S1, perhaps by previous infection with a related human coronavirus (*61*). Recently, stabilization of the MERS-CoV S2 domain was shown to elicit cross-reactive betacoronavirus antibodies in mice suggesting that the S2 domain has the potential to elicit broad coronavirus protection (*51*). For S2D14, a subset of the 20 S2 domain mutations computationally found to be stabilizing is surface-exposed, and four of those mutations (Q872A, S929K, S937G, and A1070T) are shared between all seven designs that were tested for antigenicity and may play a role in the loss of binding to VRC-112. The extent of the effect on other S2 domain epitopes and whether they can be recovered without compromising protein expression and stability is unknown and should be investigated in future studies. Despite the loss of this epitope, immunization with S2D14 resulted in high levels of neutralizing antibody titers. One possible explanation for this could be that, by inadvertently knocking out this epitope, the immune response is more focused toward the highly neutralizing epitopes on the RBDs. EM-based polyclonal epitope mapping, which has described the antibody landscape for other vaccine targets such as HIV-1 envelope (*80*–*82*) and influenza HA (*83*), has recently been applied to characterize antibody specificities against the spike protein in SARS-CoV-2 convalescent sera (*84*) and could be used to provide a detailed molecular map of the immune response elicited by S2D14. The role of T cell immunity is another important consideration for optimized S antigens which is suggested to play a role in limiting severe-to-critical COVID-19 (*85*). S-6P was shown to induce higher frequencies of antigen-specific CD8^+^ T cells producing T helper 1 cytokines than S-2P, while trimeric RBD linked to the HR1 and HR2 domain of S2 induced RBD-specific interleukin-4 and interferon-γ–producing memory T cells (*86*, *87*). Although, for this work, we only measured the humoral response as the primary way to evaluate the immunogenicity of S2D14, future studies should aim to examine the impact of mutations in S2D14 on the overall T cell response.

A major finding of this work is that although vaccination of mice with either S2D14 or S-2P results in the elicitation of similar IgG titers, S2D14 elicits a higher functional response that was more effective than S-2P in neutralizing the ancestral Wuhan strain and VoCs (Alpha, Beta, and Delta). A recent report of the immunogenicity of recombinant VSV vectored S-6P found that the enhanced stability and expression of S-6P led to the elicitation of a more effective nAb response at lower doses than S-2P against the Wuhan strain and VoC including Alpha (B.1.1.7), Gamma (P.1), Beta (B.1.351), Epsilon (B.1.427), and Delta (B.1.617.2) strains (*86*). Combined with enhanced expression, vaccination with S2D14 not only could translate well into dose-sparing effects when delivered in a recombinant setting but also may translate to a lower dose when delivered by alternative platforms such as mRNA or replication-competent viral vectors. For adjuvanted protein-based vaccines, the ability to increase expression yields of recombinant antigens would also be advantageous by providing a more cost-effective manufacturing process. Regarding the neutralization of the Omicron strain, immunization of mice with the 0.3-μg dose of S2D14 resulted in reduced nAbs when compared to titers observed against the Wuhan strain but was ~10-fold greater than titers elicited by S-2P. Therefore, S2D14 could serve as a scaffold for additional modifications which further improve nAb responses against Omicron or future variants. Overall, this work highlights the benefits of using an evolutionary consensus approach for antigen design and supports additional investigation into S2D14 as a potential tool for translational research and as a scaffold for further design and use as an immunogen for combating future coronaviruses.

## MATERIALS AND METHODS

### Computational design

Rosetta comparative modeling (RosettaCM) (*88*) was used to build a model, with cyclic symmetry constraints (*55*), of the SARS-CoV-2 S antigen with the RBD in the open conformation (PDB accession numbers: 6VSB and 6VYB). The model reconstruction strategy used a combination of x-ray and cryo-EM structures [PDB accession numbers: 6VYB, 6VW1, and 6NB7 (SARS-CoV-1)]. The symmetric interface design was performed on the lowest energy RosettaCM trimer, focusing on a monomeric chain with two virtual and identical partners, adapting protocols from the PROSS, with the updated beta energy scoring function (*49*, *50*).

To design mutations of the spike protein from SARS-CoV-2 using evolutionary constraints for the introduction of stabilizing residues, homologous sequences were obtained from the nonredundant BLAST database (*54*) and narrowed to 500 glycoprotein sequences from various coronavirus lineages (data S1). These aligned sequences were calculated into a position-specific scoring matrix (PSSM) with the PSI-BLAST algorithm (*54*). The matrix represents the likelihood for each of the 20 amino acids being present at each residue position, within the aligned sequences.

The Rosetta FilterScan mover (*89*) was used to perform single-point mutagenesis of all the residues to the preferred PSSM mutations, targeting the full spike (S) ectodomain, NTD plus S2 domain (NTD + S2), or the S2 domain only. The mutation scan was binned within 12 different energy thresholds (−0.5, −1, −1.5, −2, −2.5, −3, −3.5, −4, −4.5, −5, −5.5, and −6 kcal/mol) to increase mutation sequence diversity. A RosettaScripts (*90*) algorithm that energetically combined the proposed single mutations was used to reduce the search space onto a single monomer which was replicated across the identical protomers, yielding 12 total stabilizing designs for each round of mutations and representing each energy threshold.

### S-2P and spike mutant expression and quantification

The gene encoding the sequence of designed S mutants was synthesized and cloned into an in-house mammalian expression vector pBW, with a hexahistidine sequence added to the C terminus. For the design of the S-2P–D614G variant, cloning was performed according to the protocol in the Agilent Quickchange II Kit using the S-2P backbone as a template. The spike designs were expressed in HEK293 cells using 3.0 ml of Expi293 expression in each well of a 24-deep-well high-throughput expression system. Each design was tested in duplicate wells. Cell culture supernatants were harvested on days 5 to 6 when viability was approximately 50%.

The Octet quantification assay for protein expression level was performed on an Octet 96 Red system (Sartorius). Harvested cell media were centrifuged and cell supernatants were prepared in a 96-well plate with S-2P or S-6P standards diluted in media from 20 to 0.3125 μg/ml. The standard and mutant binding curves were measured using anti-polyhistidine biosensors (anti-HIS), where the concentration of each mutant in media was calculated by fitting the measured initial binding rate to the calibration curve. The expression levels were measured in duplicate wells of each mutant’s media and the average readout was reported.

### Purification of mutants

The culture supernatant of selected SARS-CoV-2 S mutants produced in a 1-liter scale was directly loaded through 5 ml of nickel–nitrilotriacetic acid Excel column (Cytiva Life Sciences). The column was washed with 300 mM sodium chloride and 50 mM imidazole in 20 mM Hepes buffer (pH 7.5) and captured S mutants were eluted with 300 mM sodium chloride and 250 mM imidazole in 20 mM Hepes buffer (pH 7.5). The collected samples from appropriate elution fractions were pooled and concentrated before further purification of the S trimer by gel filtration using a Superose 6 increase 10/300 GL column (Cytiva Life Sciences) with 1× phosphate-buffered saline (PBS), pH 7.4, as a running buffer. The fractions corresponding to the targeted S mutants based on SDS–polyacrylamide gel electrophoresis analysis were pooled together and quantitated using the absorbance at 280 nm.

### Surface plasmon resonance

SPR experiments were performed in a running buffer composed of 0.01 M Hepes (pH 7.4), 0.15 M NaCl, 3 mM EDTA, and 0.005% v/v surfactant P20 at 25°C using the Biacore 8K (GE Healthcare), with a series S protein A sensor chip (GE Healthcare). The ACE2 receptor or SARS-CoV-2 spike-specific antibodies (CR3022 or S309) were immobilized on the protein A sensor chip (GE Healthcare) at a ligand capture level of ~100 RU. Serial dilutions of purified spike designs were injected, at concentrations ranging from 10 to 1.25 nM. The resulting data were fit to a 1:1 binding model using the Biacore Evaluation Software (GE Healthcare).

### Differential scanning fluorimetry

Nano-DSF was used to assess the thermal stability of purified spike designs on a Prometheus NT.48 instrument (NanoTemper Technologies). Samples were diluted to 0.2 mg/ml with PBS and 20 μl of each sample was loaded into capillary tubes. A temperature ramp was set to 1°C/minute, ranging from 20° to 95°C. The reported values are the mean of the first derivative of the ratio of intrinsic tryptophan fluorescence emission wavelengths for protein unfolding/folding (350 nm/330 nm), measured in triplicate.

### Biolayer interferometry binding assays

Octet binding assays were performed using the Octet RH96 system (Sartorius). Freshly purified S-2P, S-2P–D614G, S2D14, and S-6P at 10 μg/ml were loaded onto 16 HIS1K biosensors before being dipped into 200 nM CR3022 mAb or hACE2 (Sino Biological). Immobilized antigen was allowed to associate with hACE2 or CR3022 for 10 min to achieve a signal plateau before a 10-min dissociation phase. The maximum binding signal of CR3022 and hACE2 was measured in triplicate.

### Mouse immunization studies

An in vivo study was performed to assess the immunogenicity of S2D14 compared to S-2P in a mouse model. Female BALB/c mice, 7 to 8 weeks of age at the start of the study, were immunized (*N* = 10 mice per group) with AS03-adjuvanted (an oil-in-water emulsion adjuvant system containing 1.186 mg of alpha-tocopherol per dose) spike proteins at two dosage levels of 3.0 or 0.3 μg (*91*). A saline placebo control group was also included in the study (*N* = 4) but was not considered for statistical analysis. Spike proteins and AS03 were admixed shortly before injection. Mice were injected intramuscularly twice 3 weeks apart and bled 3 weeks after the initial immunization (post-I) and 2 weeks after the second immunization (post-II).

The in vivo study was conducted in accordance with the GSK Policy on the Care, Welfare and Treatment of Laboratory Animals and was reviewed by the Institutional Animal Care and Use Committee by the ethical review process at the institution where the work was performed. All studies followed ARRIVE (Animal Research: Reporting of In Vivo Experiments) Guidelines as applicable and were conducted in compliance with provisions of the United States Department of Agriculture Animal Welfare Act, the Public Health Service Policy on Humane Care and Use of Laboratory Animals and the U.S. Interagency Research Animal Committee Principles for the Utilization and Care of Research Animals.

### Neutralization assays and ELISA

The serum CoV2-specific antibody responses were assessed using a Wuhan strain pseudo-virus neutralization assay to measure functional antibodies as described previously (*63*) and an ELISA (prefusion S-2P antigen absorbed to the solid phase) to measure IgG binding antibodies for all mice (Nexelis, Laval, Quebec). Human SARS-CoV-2 infection/COVID-19 convalescent sera (*n* = 22) were obtained at CHU Tivoli, Belgium, from donors 23 to 61 years of age, mostly from females, after polymerase chain reaction–confirmed diagnosis and at least 28 days after the participants were asymptomatic. Human samples were obtained with informed consent. All recruitment, sample collection, and experimental procedures using human samples have been approved by relevant institutional review boards and by GSK human sample management board. nAbs in post-II serum samples from mice immunized with S-2P, and S2D14 or placebo were additionally measured using a pseudo-virus neutralization assay with the Alpha, Beta, Delta, and Omicron variant strains (Nexelis, Laval, Quebec).

### Statistical analysis of IgG binding and neutralization data

To assess differences between S-2P and S2D14 vaccines, for IgG binding titers, an analysis of variance (ANOVA) model for repeated measures was fitted on log_10_-transformed data including vaccine, dose, time, and their interaction as fixed factors and considering homogeneity of variances between groups. For post-II neutralization data, except with the Omicron strain, an ANOVA model was fitting on log_10_-transformed data with vaccine and dose as fixed factors. For the Omicron assay, a model accounting for left censored data was fitted on log_10_-transformed data. Homogeneity of variances between study groups was considered for all except Alpha and Omicron assays.

Geometric mean titers (GMTs) with corresponding 95% confidence intervals and geometric mean ratios (GMRs) with 90% confidence intervals (two-sided test with alpha = 0.05) were computed from these models to compare responses to S-2P and S2D14 vaccines by dose. GMRs for which the confidence interval does not include 1 are considered statistically significant. The analyses included more vaccination groups than reported (14 for IgG data and 6 for neutralization data) as the in vivo study initially consisted of additional vaccinated groups irrelevant to this study, but a multiplicity of comparisons was not considered. GMTs with corresponding 95% confidence intervals for HCS samples were computed separately from vaccination groups using Prism (GraphPad Software, San Diego, California USA).

### Cryo-EM sample preparation

SARS-CoV-2 S designs 9 and S2D14, previously isolated by nickel-affinity chromatography, were further purified by size exclusion chromatography using a Superose 6 10/300 GL column (Cytiva Life Sciences) with tris-buffered saline buffer composed of 10 mM tris (pH 7.5) and 150 mM NaCl. Fractions containing purified spike protein were diluted to a final concentration of 0.4 mg/ml before specimen preparation on EM grids. Prepared samples (3.0 μl) were then applied to glow-discharged Quantifoil 1.2/1.3-400 mesh copper grids, blotted, and plunged into liquid ethane using an FEI Vitrobot Mark IV vitrification apparatus set at 100% relative humidity and 4°C (Thermo Fisher Scientific).

### Cryo-EM image processing and modeling

#### Glacios TEM

A total of 1425 movies were collected using an FEI Glacios TEM at 200 kV equipped with a Falcon 3 direct electron detector at a magnification of ×120,000 corresponding to a pixel size of 0.91 Å/pixel. A more detailed description of imaging parameters used during data collection can be found in Table 2.

Cryo-EM single-particle analysis was carried out using the RELION 3.1 image processing suite (*92*). Briefly, frame alignment was performed using RELION’s implementation of MotionCor2 followed by contrast transfer function (CTF) estimation using CTFFIND4.1 (*93*, *94*). A subset of images was used to manually select particles to build a set of 2D templates for automated particle picking across the entire dataset. Using the 2D templates for auto-picking, a total of 92,933 particles were selected from the full set of images. Particles were next subjected to 2D classification, which resulted in the selection of 44,459 particles. Next, 3D classification was performed against a reference structure built using RELION’s de novo 3D model generation and applying C3 symmetry. Three classes retained the trimeric morphology, and the fourth class was discarded because of poor structure quality. The three classes consisting of 39,718 particles were subjected to an additional round of 3D classification using C1 symmetry, which resulted in one class of particles with three RBDs in a closed state (7759 particles) and a second class with one RBD in the open state (9154 particles).

For particles in the closed conformation, 3D refinement was performed using C3 symmetry, while C1 symmetry was used for 3D refinement for the one-RBD open conformation. After initial 3D refinement, both datasets were followed by CTF refinement to correct for beam aberrations and determine per-particle defocus values. Bayesian polishing of the CTF-refined particles was then performed to correct for per-particle motion. A final 3D refinement of both structures converged to 7.1 Å for the RBD closed conformation and 8.5 Å for the single RBD open conformation according to the gold-standard 0.143 Fourier shell correlation (FSC) criteria.

#### Titan Krios

A total of 6169 movies were collected using an FEI Titan Krios operating at 300 kV and equipped with a Falcon 4 direct electron detector (Nanosciences Center, Cambridge University, UK) at a magnification of ×120,000 corresponding to a pixel size of 0.67 Å/pixel. A more detailed description of settings used for imaging during data collection can be found in Table 2.

Cryo-EM single-particle analysis was carried out using the RELION 3.1 image processing suite (*92*). For a visual depiction of the single-particle workflow, refer to fig. S7. Briefly, alignment of the raw movie frames was performed using RELION’s implementation of MotionCor2 followed by CTF estimation using CTFFIND4.1 (*93*, *94*). Next, a subset of particles was manually selected and used to generate 2D classes that were then used as templates for particle-picking across the entire set of 6169 micrographs, which resulted in the selection of 724,002 particles. Particles were initially extracted at 4× binning with a pixel size of 2.68 Å/pixel. The binned particle stack was subjected to a single round of 2D classification and classes showing that features consistent with the S trimer were selected for further processing. The 566,825 particles selected from 2D classification were subjected to one round of 3D classification against a reference model that was generated using RELION’s de novo 3D model generation. Among the generated classes was a single population resembling the known trimeric S morphology. This stack of 205,674 particles was then re-extracted as the unbinned particle set. A consensus C1 refinement was performed to estimate correct particle poses resulting in a 3.8-Å resolution cryo-EM map. The focused 3D classification was then performed using individual masks encompassing the NTD and RBD for RBDs in the closed state, one-RBD open, and two-RBD open conformations, which resulted in the separation of the particles into three distinct classes: a three-RBD closed class (33,173 particles), a two-RBD exposed class (47,773 particles), and a two-RBD open class (87,614 particles). Initial 3D auto-refinement for the three-RBD closed, two-RBD exposed, and two-RBD open conformations resulted in 3.9-, 3.5-, and 3.5-Å resolution cryo-EM maps, respectively. Three-dimensional refined particles for each set were further subjected to CTF refinement and Bayesian polishing to estimate per-particle defocus values and correct for individual particle motions. A final 3D refinement resulted in an improved resolution for each map according to the gold-standard 0.143 FSC criteria. Final resolutions for the three-RBD closed, two-RBD exposed, and two-RBD open conformations were 2.8, 3.3, and 3.1 Å, respectively.

Modeling of each cryo-EM structure was performed using Phenix and COOT (*95*, *96*). For the three-RBD closed conformation, PDB 6VXX was used as a starting structure where mutations for S2D14 were inserted using ChimeraX (*17*, *97*). For the RBD exposed and RBD open structures, a model for the two-RBD open conformation was built from PDB 6VSB, and mutations for S2D14 were inserted using ChimeraX (*20*). For each model and corresponding map, real-space refinement was performed in Phenix, initially by applying morphing, simulated annealing, and applying secondary structure restraints. To rebuild loops missing in the original PDB files and fit into the corresponding cryo-EM density, RosettaCM was performed using the initial Phenix models and structural templates for known NTD and RBD structures (PDB accession numbers: 7LY3 and 7DEU). Once the full homology model was built, Rosetta Cartesian refinement was performed (*98*), followed by torsional refinement to build glycan structures (*99*, *100*). A final real-space refinement applying minimization, atomic displacement parameter refinement, and occupancy optimization was performed using Phenix. For the RBD exposed and RBD open structures, 0.14 and 0.03% of their residues were classified in the disallowed regions of Ramachandran space, but models were not further adjusted because of poor map quality in these regions. Loops, disordered regions, and glycans lacking structural information in the cryo-EM density were removed in the resulting Rosetta models.

## Notes

View/request a protocol for this paper from *Bio-protocol*.
