# Supplementary material for: Structural and computational design of a SARS-CoV-2 spike antigen with improved expression and immunogenicity
Source: Sci Adv. 2023 Jun 7;9(23):eadg0330. doi: 10.1126/sciadv.adg0330 (PMC10246912; doi:10.1126/sciadv.adg0330)
Supplement: Supplementary file 1 — Figs. S1 to S11 Table S1 Legends for data S1 and S2 [file sciadv.adg0330_sm.pdf]

Science Advances
AAAS

Supplementary Materials for

Structural and computational design of a SARS-CoV-2 spike antigen with improved expression and immunogenicity

James A. Williams *et al.*

Corresponding author: Newton Wahome, newton@wahome.org;
Wayne D. Harshbarger, wayne.d.harshbarger@gsk.com

Sci. Adv. **9**, eadg0330 (2023)
DOI: 10.1126/sciadv.adg0330

The PDF file includes:

Figs. S1 to S11
Table S1
Legends for data S1 and S2

Other Supplementary Material for this manuscript includes the following:

Data S1 and S2

A

Full S-Ectodomain		
**CoV S Mutant**	**Selected Design**	**Rosetta score (kcal/mol)**
Target sequence		-11615.951
PreS_S_0_5		-12405.366
PreS_S_1	1	-12339.751
PreS_S_1_5		-12294.3
PreS_S_2	2	-12315.109
PreS_S_2_5		-12278.535
PreS_S_3		-12251.713
PreS_S_3_5	3	-12213.033
PreS_S_4		-12192.985
PreS_S_4_5		-12159.976
PreS_S_5	4	-12080.037
PreS_S_5_5		-12056.763
PreS_S_6	5	-12064.728

Full S-Ectodomain

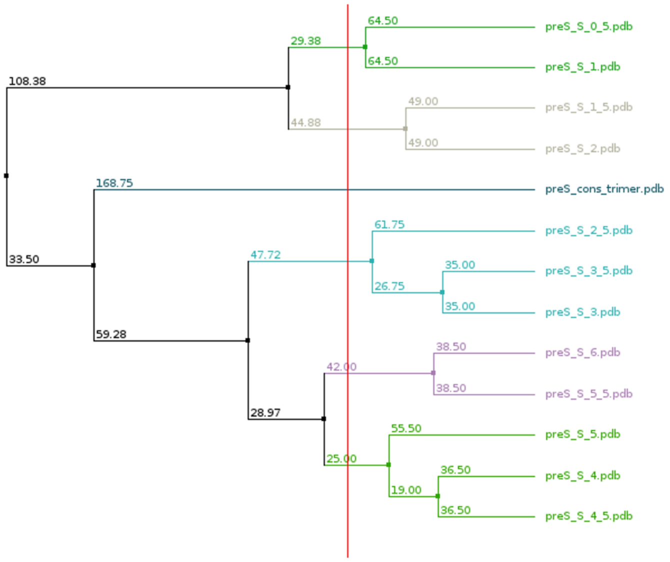

B

NTD + S2		
**CoV S Mutant**	**Selected Design**	**Rosetta score (kcal/mol)**
Target sequence		-11615.951
PreS_S2_NTD_0_5	6	-12142.196
PreS_S2_NTD_1		-12202.403
PreS_S2_NTD_1_5		-12213.696
PreS_S2_NTD_2	7	-12192.697
PreS_S2_NTD_2_5		-12159.516
PreS_S2_NTD_3	8	-12139.76
PreS_S2_NTD_3_5		-12078.789
PreS_S2_NTD_4		-12064.461
PreS_S2_NTD_4_5		-12065.383
PreS_S2_NTD_5	9	-12028.133
PreS_S2_NTD_5_5		-11998.548
PreS_S2_NTD_6	10	-11957.319

NTD + S2 domain

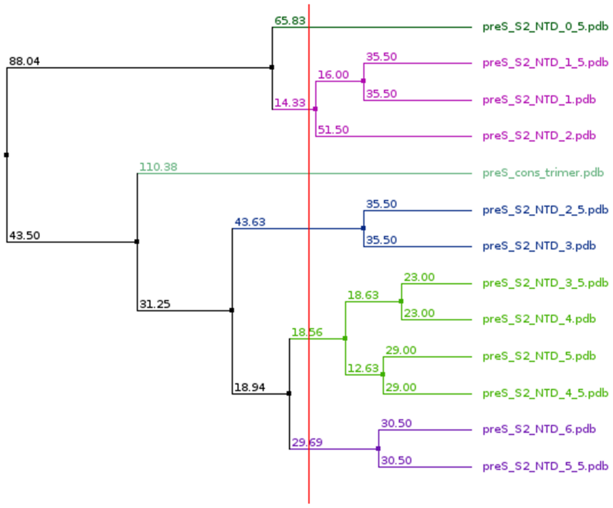

C

S2 Domain		
CoV S Mutant	Selected Design	Rosetta score (kcal/mol)
Target sequence		-11615.951
PreS_S2_0_5		-12072.375
PreS_S2_1	11	-12028.056
PreS_S2_1_5		-12031.222
PreS_S2_2	12	-12040.859
PreS_S2_2_5		-12035.513
PreS_S2_3	13	-11985.224
PreS_S2_3_5		-11988.799
PreS_S2_4	14	-11969.559
PreS_S2_4_5		-11922.035
PreS_S2_5		-11963.851
PreS_S2_5_5		-11928.956
PreS_S2_6	15	-11909.424

S2 domain

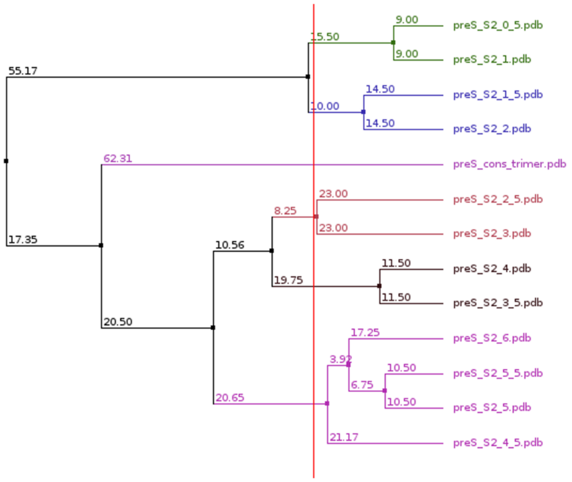

Fig. S1. Rosetta energy scoring and selection of Spike protein designs. Phylogenetics using BLOSUM62 sequence analysis matrix in JalView and the average distance algorithm was used for each domain-specific design strategy to separate sequences into five groups (indicated by color and excluding the preS cons trimer). Since members of each group were highly similar, a single representative from each group was randomly selected, resulting in a total of 15 sequences for analysis. **(A)** Rosetta energy scores for the full S-ectodomain designs and selection of five designs, 1-5, using phylogenetics (right). **(B)** Rosetta energy scores for NTD + S2 domain designs and selection of five designs, 6-10, using phylogenetics (right). **(C)** Rosetta energy scores for S2 domain designs and selection of five designs, 11-15 using phylogenetics (right). Rosetta energy calculations for all designs were predicted to be more stable than the prefusion Spike target sequence. *preS_cons trimer (target sequence) is the S model built from multiple experimental structures and containing S-2P di-proline (K986P and V987P), and the D614G drift mutations.

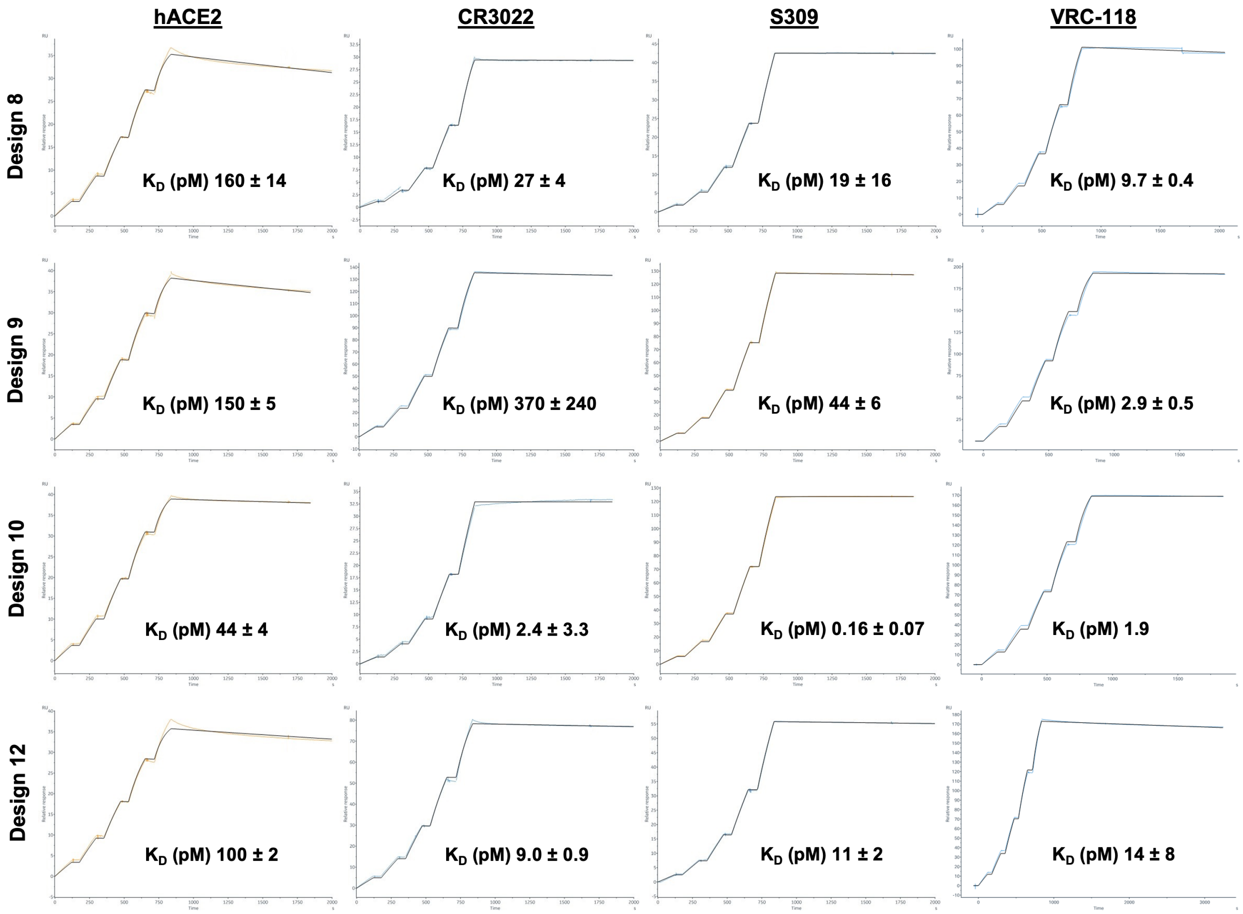

hACE2
CR3022
S309
VRC-118
Design 8
Design 9
Design 10
Design 12
K_D (pM) 160 ± 14
K_D (pM) 27 ± 4
K_D (pM) 19 ± 16
K_D (pM) 9.7 ± 0.4
K_D (pM) 150 ± 5
K_D (pM) 370 ± 240
K_D (pM) 44 ± 6
K_D (pM) 2.9 ± 0.5
K_D (pM) 44 ± 4
K_D (pM) 2.4 ± 3.3
K_D (pM) 0.16 ± 0.07
K_D (pM) 1.9
K_D (pM) 100 ± 2
K_D (pM) 9.0 ± 0.9
K_D (pM) 11 ± 2
K_D (pM) 14 ± 8

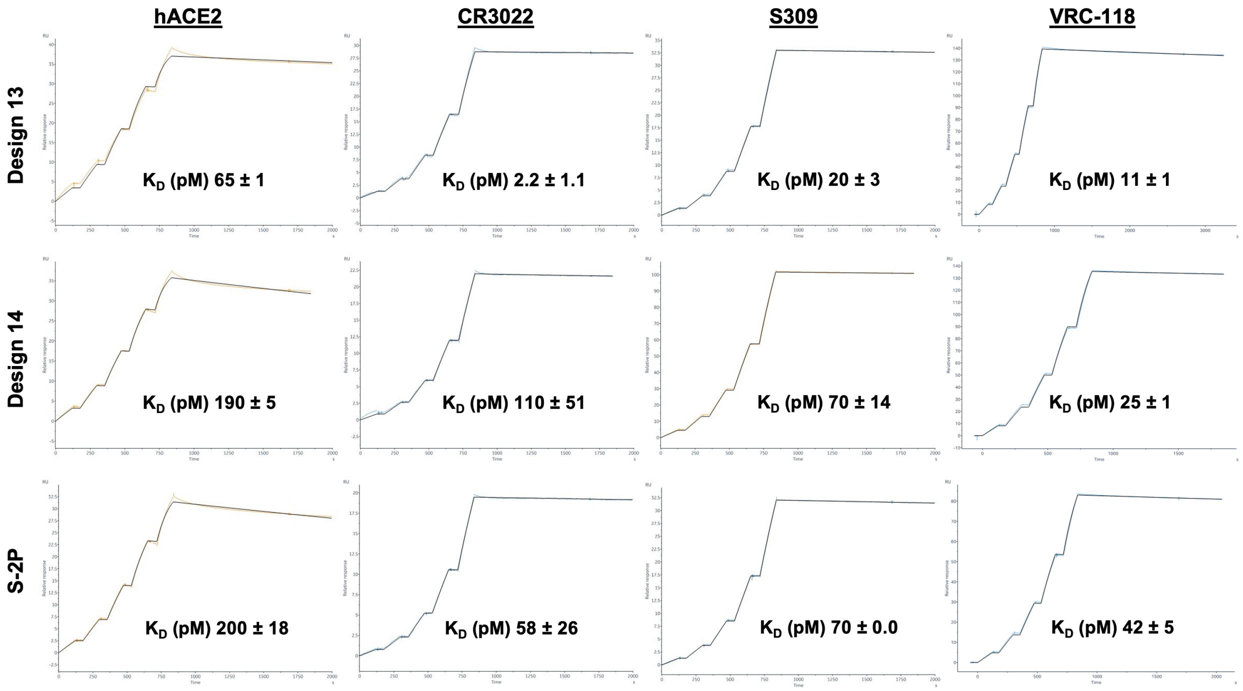

Fig. S2. Selected SARS-CoV-2 S designs and S-2P are capable of binding hACE2 receptor, CR3022, S309, and VRC-118 (NTD). SPR binding curves for selected S designs and S-2P. Data were fit to a 1:1 binding model (calculated fit is shown as black line) and the average K_D and standard deviations for triplicate measurements are shown below each binding curve. Binding of design 10 to VRC-118 was limited to a single measurement due to limited protein quantity. See table S1 for kinetic parameters.

Antigen	Receptor/Binding antibody	k_a (1/Ms)	k_d (1/s)
S-2P	ACE2	$4.9x10^{+5}±2.5x10^{+4}$	$1.0x10^{-4}±5.3x10^{-6}$
	CR3022	$2.3x10^{+5}±7.4x10^{+4}$	$1.2x10^{-5}±2.9x10^{-6}$
	S309	$2.0x10^{+5}±1.5x10^{+4}$	$1.4x10^{-5}±1.2x10^{-6}$
	VRC-118	$5.7x10^{+5}±6.5x10^{+3}$	$2.3x10^{-5}±2.5x10^{-6}$
8	ACE2	$5.9x10^{+5}±2.7x10^{+4}$	$1.0x10^{-4}±4.1x10^{-6}$
	CR3022	$2.3x10^{+5}±3.1x10^{+4}$	$6.5x10^{-6}±1.2x10^{-9}$
	S309	$2.7x10^{+5}±3.9x10^{+4}$	$5.6x10^{-6}±5.1x10^{-6}$
	VRC-118	$6.8x10^{+5}±6.6x10^{+1}$	$6.6x10^{-6}±2.6x10^{-7}$
9	ACE2	$5.7x10^{+5}±2.3x10^{+4}$	$8.9x10^{-5}±2.9x10^{-6}$
	CR3022	$1.5x10^{+5}±6.4x10^{+3}$	$5.5x10^{-5}±3.4x10^{-5}$
	S309	$1.8x10^{+5}±3.0x10^{+3}$	$8.0x10^{-6}±1.1x10^{-6}$
	VRC-118	$1.0x10^{+6}±2.2x10^{+4}$	$3.2x10^{-6}±5.0x10^{-7}$
10	ACE2	$6.2x10^{+5}±1.3x10^{+3}$	$2.7x10^{-5}±2.6x10^{-6}$
	CR3022	$3.4x10^{+5}±1.7x10^{+5}$	$1.1x10^{-6}±1.5x10^{-6}$
	S309	$1.8x10^{+5}±6.5x10^{+3}$	$3.0x10^{-8}±1.4x10^{-8}$
	VRC-118	$9.2x10^{+5}$	$1.8x10^{-6}$
12	ACE2	$6.2x10^{+5}±3.6x10^{+3}$	$6.3x10^{-5}±8.7x10^{-7}$
	CR3022	$1.7x10^{+6}±2.3x10^{+4}$	$1.5x10^{-5}±1.3x10^{-6}$
	S309	$8.4x10^{+5}±6.8x10^{+4}$	$9.4x10^{-6}±7.1x10^{-7}$
	VRC-118	$1.9x10^{+6}±3.5x10^{+4}$	$2.7x10^{-5}±1.5x10^{-5}$
13	ACE2	$6.1x10^{+5}±1.0x10^{+4}$	$4.0x10^{-5}±2.3x10^{-7}$
	CR3022	$1.0x10^{+6}±5.4x10^{+4}$	$2.3x10^{-6}±1.0x10^{-6}$
	S309	$5.0x10^{+5}±7.8x10^{+4}$	$1.0x10^{-5}±2.4x10^{-6}$
	VRC-118	$1.5x10^{+6}±3.3x10^{+4}$	$1.8x10^{-5}±9.6x10^{-7}$
14	ACE2	$5.8x10^{+5}±1.8x10^{+3}$	$1.1x10^{-4}±2.6x10^{-6}$
	CR3022	$2.1x10^{+5}±4.4x10^{+3}$	$2.4x10^{-5}±1.1x10^{-5}$
	S309	$1.5x10^{+5}±3.2x10^{+3}$	$1.0x10^{-5}±2.0x10^{-6}$
	VRC-118	$6.3x10^{+5}±8.8x10^{+3}$	$1.6x10^{-5}±5.4x10^{-7}$

Table S1: Kinetic parameters for the binding of hACE2, RBD targeting antibodies CR3022 and S309, and NTD targeting antibody VRC-118 to selected SARS-CoV-2 S designs. Shown are the average and standard deviation for triplicate measurements.

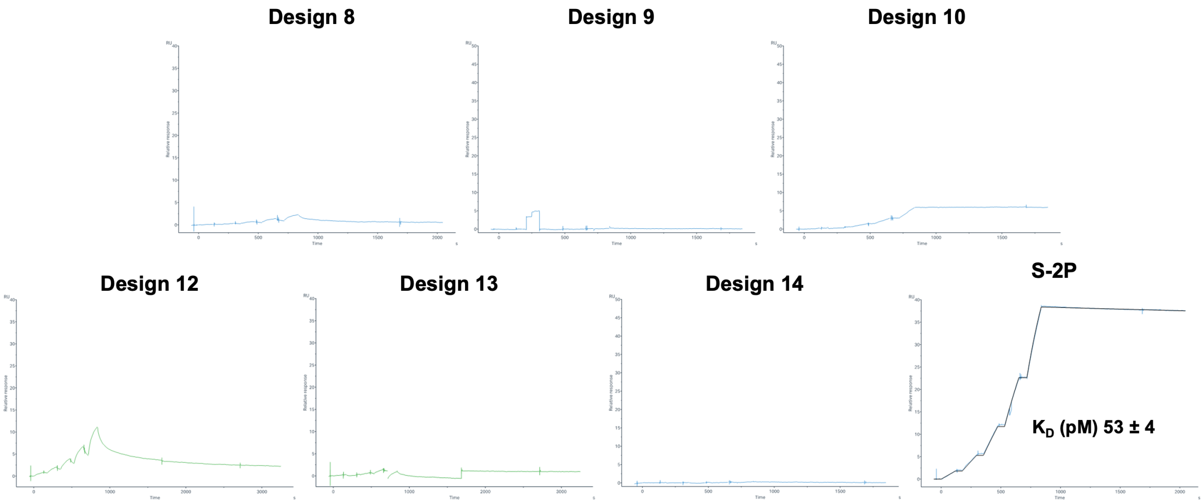

Fig. S3. The VRC-112 (S2 domain) epitope is disrupted for SARS-CoV-2 S designs. SPR binding curves for selected S designs and S-2P. Data were fit to a 1:1 binding model (calculated fit is shown as black line) and the average K_D and standard deviation for triplicate measurements are shown for S-2P.

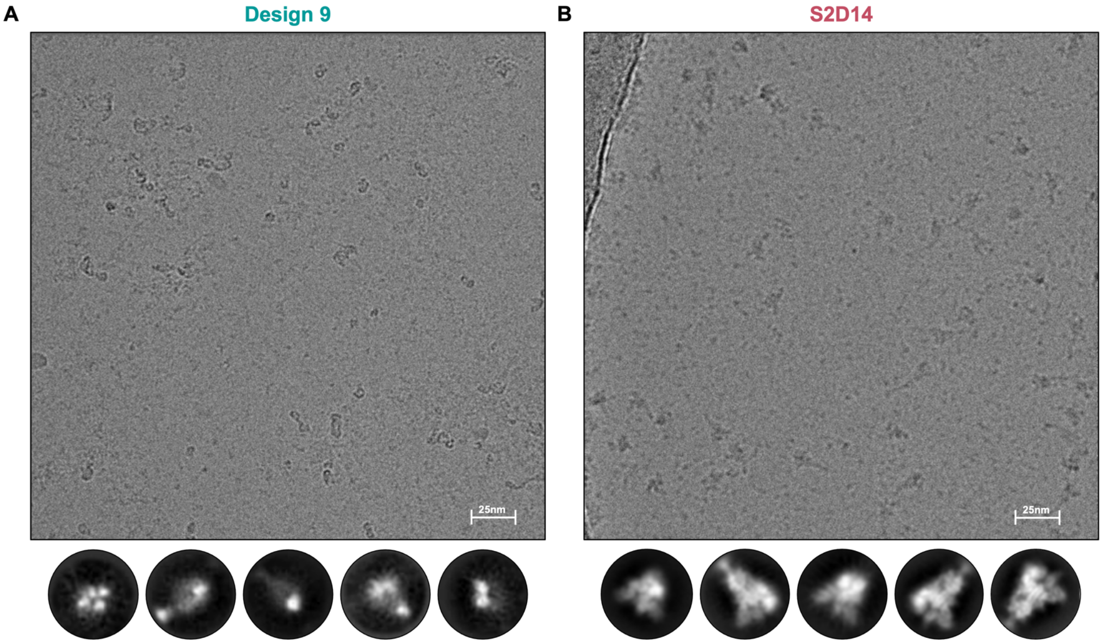

Fig. S4. Cryo-EM screening of design 9 and S2D14 (design 14) confirms that only S2D14 forms stable prefusion S trimers. **(A)** Micrograph and 2D class averages (below) of design 9 illustrating the lack of observable S protein trimers. **(B)** In contrast, images collected for S2D14 contained particles that were consistent with the expected morphology and size of S trimers. 2D classes of S2D14 from Fig. 2 are shown below for comparison. Scale bar = 25 nm

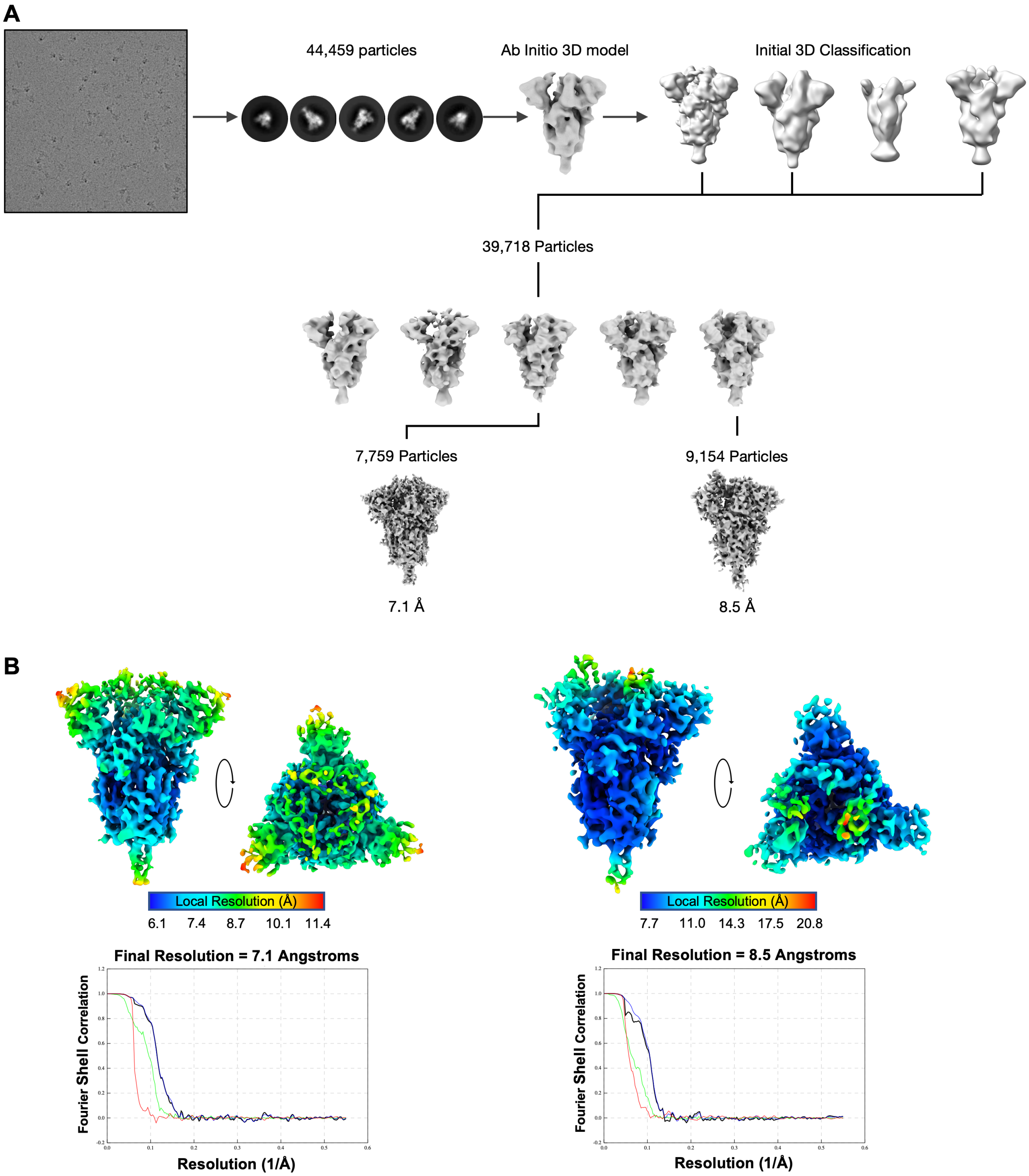

Fig. S5. Single particle reconstruction workflow. (A) Schematic illustrating the cryo-EM data processing workflow for images collected on a Glacios TEM and using RELION 3.1 image processing suite. **(B)** Local resolution plots were generated for each map using RELION 3.1 and the FSC curves (below) report the resolution using the gold-standard FSC at 0.143 criteria.

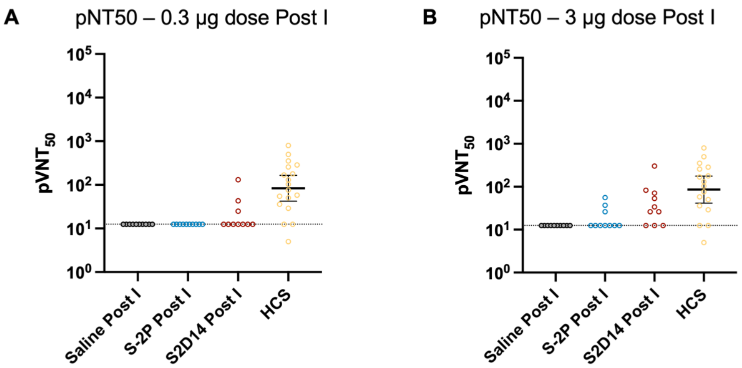

Fig. S6. Neutralizing antibody titers from immunized mice against Wuhan post-I compared to human convalescent serum. Neutralizing antibody titers against the Wuhan strain were analyzed 3 weeks post-I vaccination at two antigen dosages, 0.3 µg **(A)** and 3.0 µg **(B)**. Neither S-2P or S2D14 elicited a neutralizing response post-I compared to human convalescent sera (HCS). For HCS samples, GMT and 95% CI were calculated separately from vaccination groups in Prism GraphPad. See Methods for a description of the sera panel.

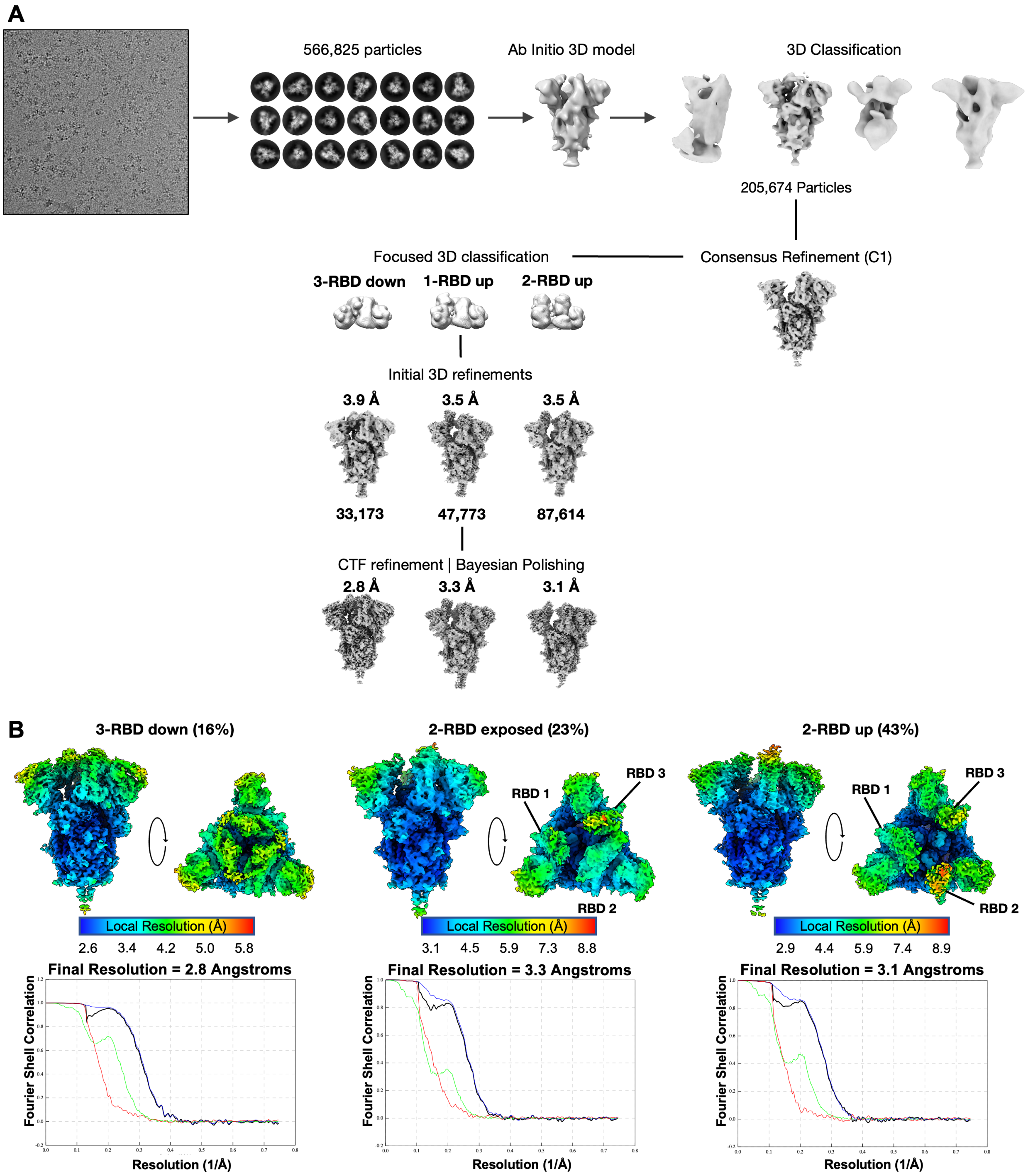

Fig. S7. High-resolution single particle reconstruction workflow. (A) Schematic illustrating the cryo-EM data processing workflow for images collected on a Titan Krios and using RELION 3.1 image processing suite. **(B)** Local resolution plots were generated for each map using RELION 3.1 and the FSC curves (below) report the resolution using the gold-standard FSC at 0.143 criteria.

A **hACE2**

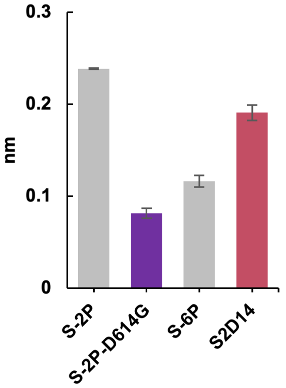

hACE2 Binding	S-2P	S-2P D614G	S-6P	S2D14
R1 – Binding level (nm)	0.2391	0.837	0.1097	0.1938
R2 – Binding level (nm)	0.2385	0.0852	0.1225	0.1971
R3 – Binding level (nm)	0.2379	0.0752	0.1159	0.1813
Mean (nm)	0.2385	0.0813	0.1160	0.1813
Std. Deviation (± nm)	0.0006	0.0053	0.0064	0.0083
Response (% to S-2P)	100	34	49	80

B **CR3022**

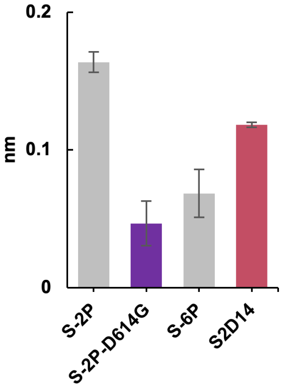

CR3022 Binding	S-2P	S-2P D614G	S-6P	S2D14
R1 – Binding level (nm)	0.1716	0.0288	0.0483	0.1161
R2 – Binding level (nm)	0.1619	0.0605	0.0768	0.1197
R3 – Binding level (nm)	0.1573	0.0504	0.0801	0.1184
Mean (nm)	0.1637	0.0466	0.0684	0.1181
Std. Deviation (± nm)	0.0074	0.0162	0.0175	0.0018
Response (% to S-2P)	100	28	42	72

Fig. S8. Peak binding response using BLI does not indicate greater RBD exposure for S2D14 compared to S-2P. (A) Peak binding responses of S-2P, S-2P-D614G, S-6P, and S2D14 to hACE2. The peak signal for individual measurements, mean, standard deviation and percent response compared to S-2P are reported in the Table (right) **(B)** Peak binding responses of S-2P, S-2P-D614G, S-6P, and S2D14 to CR3022. The peak signal for individual measurements, mean, standard deviation and percent response compared to S-2P are reported in the Table (right) Triplicate measurements were taken, and error bars represent standard deviation from the mean.

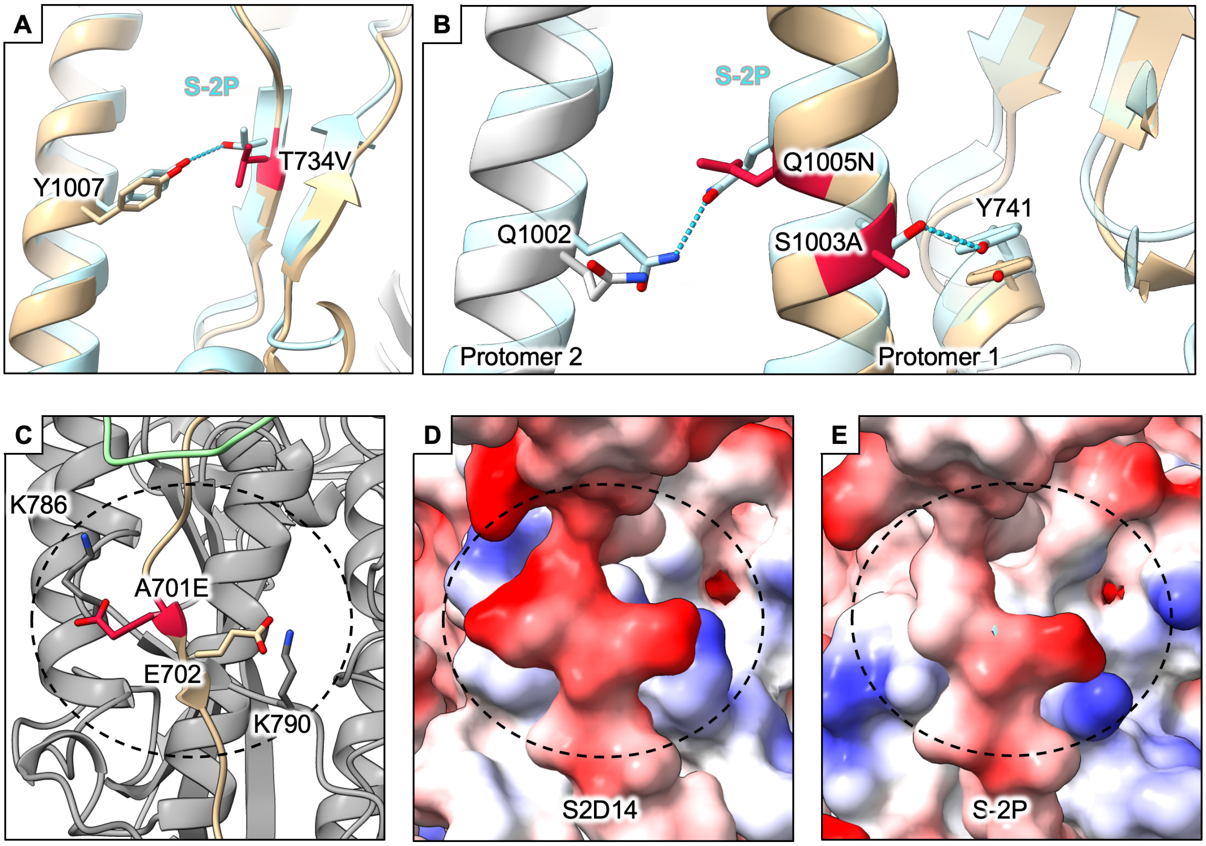

Fig. S9. Mutations within S2D14 that affect hydrogen bond formation and electrostatic interactions. Hydrogen bonds in S-2P are removed by mutations such as **(A)** T734V, and **(B)** S1003A and Q1005N**.** Hydrogen bonds are depicted as blue dotted lines. **(C)** The A701E mutation in the linker region between the S1 and S2 domain is in proximity to a positively charged lysine at position K786 on a neighboring protomer and adjacent to a salt bridge formed between E702 and K790. Comparison of the S2D14 electrostatic potential **(D)** of the S1/S2 linker to S-2P **(E)** shows a greater negative patch for S2D14 that may strengthen interprotomer contacts via complementary electrostatic interactions.

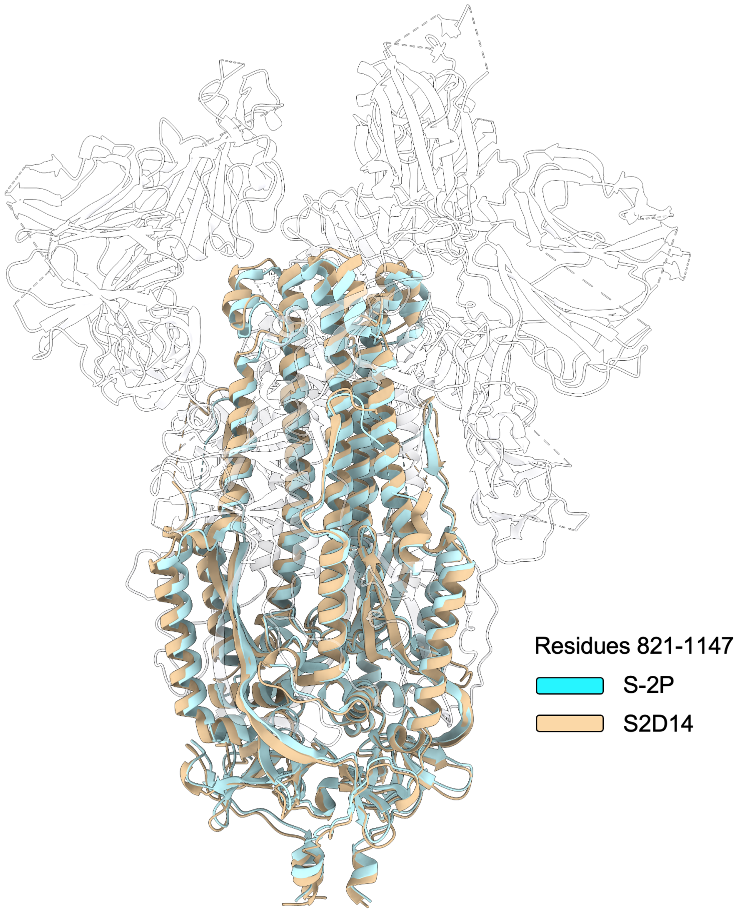

Fig. S10. S2 domain architecture for S2D14 is not disrupted by S2 domain mutations. Superposition of S-2P (PDB 6VSB) and S2D14 (two RBD open structure) S2 domain residues 821-1147 reveals an RMSD of 1.6 Å over all C_a atoms. S-2P is colored in light blue and S2D14 is colored in tan. The S1 domain of S2D14 is shown as a silhouette for clarity.

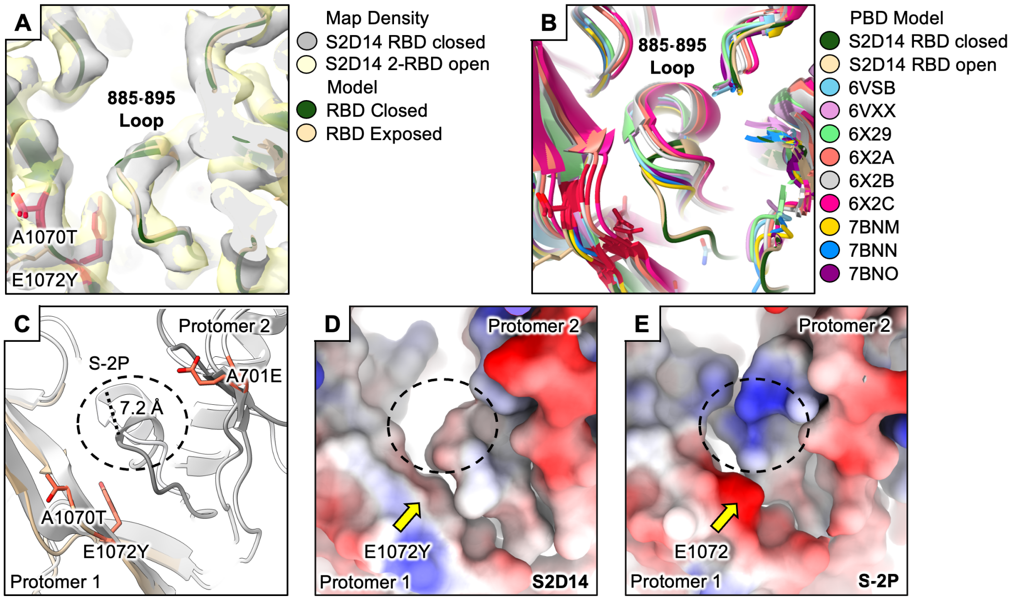

Fig. S11. Mutations within S2D14 that reposition the 885-890 loop. (A) Superposition of S2D14 structures and EM densities for the three RBD closed and two RBD open state show identical positioning of the 885-895 loop in proximity to the A1070T and T1072Y mutations. **(B)** The position of the 885-895 loop compared with several published structures shows that this loop placement is unique to S2D14. **(C)** Superposition of S-2P (light grey) and S2D14 shows an ~7.2 Å shift of the 885-895 loop towards the E1072Y mutation. **(D)** The same orientation as shown in (C) but with S2D14 depicted in electrostatic surface. The dotted circle highlights a gap in the S2D14 trimer interior caused by displacement of the 885-895 loop, which is now packed against a hydrophobic patch created by the E1072Y mutation (indicated by a yellow arrow). **(E)** The same orientation as shown in (C) but with S-2P depicted in electrostatic surface. The dotted circle highlights the position of the 885-895 loop towards the S-2P trimer interior. The wild-type E1072 is indicated by a yellow arrow.

Data S1: Sequences for the betacoronavirus Spike glycoproteins used to guide evolutionary consensus design.

Data S2: Amino acid sequences for designed Spike antigens 1 through 15.
